# Phase I Trial of Intravenous Mistletoe Extract in Advanced Cancer

**DOI:** 10.1158/2767-9764.CRC-23-0002

**Published:** 2023-02-28

**Authors:** Channing J. Paller, Lin Wang, Wei Fu, Rajendra Kumar, Jennifer N. Durham, Nilofer S. Azad, Daniel A. Laheru, Ilene Browner, Sushant K. Kachhap, Kavya Boyapati, Thomas Odeny, Deborah K. Armstrong, Christian F. Meyer, Stephanie Gaillard, Julie R. Brahmer, Ivelisse Page, Hao Wang, Luis A. Diaz

**Affiliations:** 1 The Sidney Kimmel Comprehensive Cancer Center, Johns Hopkins University School of Medicine, Baltimore, Maryland.; 2 Department of Epidemiology, Johns Hopkins Bloomberg School of Public Health, Baltimore, Maryland.; 3 Center for Cancer Research, NCI, NIH, Bethesda, Maryland.; 4 The Believe Big Institute of Health, Believe Big Inc., Cockeysville, Maryland.; 5 Division of Solid Tumor Oncology, Memorial Sloan Kettering Cancer Center, New York, New York.

## Abstract

**Purpose:**

Mistletoe extract (ME) is widely used for patients with cancer to support therapy and to improve quality of life (QoL). However, its use is controversial due to suboptimal trials and a lack of data supporting its intravenous administration.

**Materials and Methods:**

This phase I trial of intravenous mistletoe (Helixor M) aimed to determine the recommended phase II dosing and to evaluate safety. Patients with solid tumor progressing on at least one line of chemotherapy received escalating doses of Helixor M three times a week. Assessments were also made of tumor marker kinetics and QoL.

**Results:**

Twenty-one patients were recruited. The median follow-up duration was 15.3 weeks. The MTD was 600 mg. Treatment-related adverse events (AE) occurred in 13 patients (61.9%), with the most common being fatigue (28.6%), nausea (9.5%), and chills (9.5%). Grade 3+ treatment-related AEs were noted in 3 patients (14.8%). Stable disease was observed in 5 patients who had one to six prior therapies. Reductions in baseline target lesions were observed in 3 patients who had two to six prior therapies. Objective responses were not observed. The disease control rate (percentage of complete/partial response and stable disease) was 23.8%. The median stable disease was 15 weeks. Serum cancer antigen-125 or carcinoembryonic antigen showed a slower rate of increase at higher dose levels. The median QoL by Functional Assessment of Cancer Therapy-General increased from 79.7 at week 1 to 93 at week 4.

**Conclusions:**

Intravenous mistletoe demonstrated manageable toxicities with disease control and improved QoL in a heavily pretreated solid tumor population. Future phase II trials are warranted.

**Significance:**

Although ME is widely used for cancers, its efficacy and safety are uncertain. This first phase I trial of intravenous mistletoe (Helixor M) aimed to determine phase II dosing and to evaluate safety. We recruited 21 patients with relapsed/refractory metastatic solid tumor. Intravenous mistletoe (600 mg, 3/week) demonstrated manageable toxicities (fatigue, nausea, and chills) with disease control and improved QoL. Future research can examine ME's effect on survival and chemotherapy tolerability.

## Introduction

Patients with cancer experience physical impairment and a decline in quality of life (QoL) related to disease and therapy. The use of complementary medicine to support treatment and improve QoL has been increasingly popular, rising from 25% of patients in the 1970s to 50% by 2000 (1). More recent surveys have shown similar statistics and dynamic (2). *Viscum album* L., or European mistletoe has been used for decades as a complementary cancer treatment (3). It is a semiparasitic plant with active ingredients that vary by host-tree species (4). Bioactive components, including lectins, viscotoxins, polysaccharides, flavonoids, and others, enhance tumor cytotoxicity *in vitro* (5) and immunomodulation (6). Cytotoxic effects of the mistletoe extract (ME) are reported to be a result of protein synthesis interference (7, 8), cell-cycle inhibition (9), and induced apoptosis (9–12). Administration of ME has been associated with an increase in the white blood cell count and cytokines (13–15). I has also been suggested that ME has antiangiogenic properties (16). These potential antineoplastic properties of mistletoe identified in preclinical studies have not been evaluated in clinical trials.

Preparations from ME are the most frequently prescribed complementary medicine in cancer treatment in German-speaking countries, either as a sole treatment or during chemotherapy or radiotherapy (17). While ME is not approved for cancer treatment in the United States, *Viscum album* L. is listed in the U.S. Homeopathic Pharmacopoeia and is offered in integrative care clinics (18). The stated therapeutic objectives are to stimulate the immune system, improve QoL, and reduce adverse events (AE) associated with chemotherapy or radiotherapy (19). However, this treatment modality is considerably debated. A Cochrane review of randomized controlled trials (RCT) found that evidence supporting the claimed efficacy of these outcomes was weak, although some evidence suggested that ME may improve QoL (17).

Traditional ME therapy is administered subcutaneously, but local subcutaneous injection is limited because of pain and swelling at the injection site (20). To increase dose levels, “off-label” intravenous administration is used. In a retrospective study of 4,695 patients with cancer treated in Germany between 2003 and 2013, 62% received ME treatment, and 10% received it intravenously (21).

There are three types of ME, Helixor-P (pine trees), Helixor-M (apple trees), and Helixor-A (fir trees; ref. 5). Their growth inhibitory effects have been investigated in a panel of 38 human tumor cell lines *in vitro* (5). These tumor cell lines covered melanoma, lung, breast, prostate, colon, pancreatic, bladder, renal, ovarian, and uterine cancers. Helixor-P affected the most potent cytotoxic activity, followed by Helixor-M and Helixor-A with IC_50_ (50% inhibitory concentration) values of 68.4, 114, and 133 μg/mL, respectively. *In vivo* experiments on the antitumor activity of Helixor-M were performed in a BALB/c-mouse/BT474 ductal breast carcinoma model. The experiments found that tumors of Helixor-M–treated groups showed a decreased cell proliferation rate, as well as an increased cell necrosis and apoptosis rate, compared with tumors of control mice (22). Helixor-P was evaluated in a phase I trial with weekly intravenous infusion, with a dose escalation from 200 to 2,000 mg and the MTD was not reached (23). Despite Helixor-M having solid immunomodulatory effects (24) and being the most commonly prescribed ME in Europe (21), its MTD is unknown. Similarly, Helixor-A has not been studied in intravenous form to date.

In light of the preclinical cytotoxicity of Helixor-M, its widespread use, yet controversial efficacy, and especially the lack of safety data for its intravenous administration, this phase I trial (ClinicalTrials.gov identifier: NCT03051477) tested intravenous Helixor-M in patients with solid tumor to determine its safety and MTD. Tumor responses, serum cytokines, tumor markers, and QoL were also measured.

## Materials and Methods

Patients were recruited at the Johns Hopkins University Sidney Kimmel Comprehensive Cancer Center (SKCCC). Eligible adult patients (≥18 years) had advanced solid tumors and had received at least one standard systemic therapy with chemotherapy, immunotherapy, hormonal therapy, or other therapies for metastatic disease and had disease progression according to the RECIST guideline, version 1.1. Patients had an Eastern Cooperative Oncology Group (ECOG) performance status of 0, 1, or 2 and had a life expectancy longer than 3 months. Major exclusion criteria included (i) history or evidence of brain metastases, (ii) chemotherapy, radiation, hormonal therapy, or biological cancer therapy within 28 days before study treatment initiation, (iii) prior mistletoe treatment, (iv) anticipated other forms of concurrent systemic or localized antineoplastic therapy, and (v) a history of chronic autoimmune disease or chronic infection.

### Study Treatment and Dose Escalation

Helixor-M was administered intravenously on Monday, Wednesday, and Friday on a weekly basis. Four dose levels of mistletoe were evaluated: 150, 300, 600, and 900 mg. The dose frequency and starting and maximum dose levels were informed by the phase I trial of intravenous Helixor P (23). At each dose level, patients received a lead dose of 50 mg on week 1 day 1 and the assigned dose level thereafter. Patients were observed for dose-limiting toxicities (DLT) for 28 days after the first dose. DLTs are treatment-related grade 3 and above AEs defined in the trial protocol (Supplementary Materials and Methods). Treatment continued until DLTs, disease progression, or intercurrent illnesses prevented further treatment.

A traditional 3 + 3 method was employed, where dose escalation continued when at least 3 patients completed a safety evaluation at a given dose level with DLTs in fewer than a third of patients. The MTD was defined as the dose level immediately below the dose level at which 2 or more patients in a cohort (dose level) experienced a treatment-related DLT.

### Safety and Efficacy

Patients were followed for 28 days after the last dose. Thereafter, patients were contacted every 6 months to monitor overall survival (OS). Safety evaluations (clinical and laboratory examinations) were conducted every 28 days, beginning from the first dose. AEs were graded according to revised Common Terminology Criteria for AEs, version 4.03. Efficacy evaluations with imaging were conducted at baseline, week 8, and every 8 weeks thereafter, except for patients who came off trial before 8 weeks or those for whom imaging assessment was deemed necessary due to symptoms of clinical progression. Response and progression were assessed using the revised RECIST guideline, version 1.1 (for details, see the trial protocol in Supplementary Materials and Methods). QoL was measured using the Functional Assessment of Cancer Therapy-General (FACT-G) questionnaire, version 4, at baseline, week 4, and the end of treatment.

### Pharmacodynamic Analysis

Serum was collected from patients at baseline, every 4 weeks, and after treatment to identify potential therapeutic targets, biomarkers, and response predictors. Carcinoembryonic antigen (CEA) was evaluated when clinically expressed. Serum analysis for cytokine production by peripheral blood mononuclear cells was performed using a Human Cytokine/Chemokine/Growth Factor panel consisting of 38 analytes (see the trial protocol in Supplementary Materials and Methods).

### Quality

The Investigational New Drug (IND) application for this study was approved by the FDA. The study was approved by the Johns Hopkins University Institutional Review Board. All patients (or their legal representatives) gave written informed consent before enrollment. The study was conducted in accordance with the Declaration of Helsinki and the International Council for Harmonization Good Clinical Practice guidelines and monitored externally by the SKCCC Clinical Research Office and Safety Monitoring Committee.

The SKCCC was the IND sponsor of this study and was responsible for study design, data collection, analysis, and result interpretation. All the authors attest to the fidelity of trial conduct to its protocol, vouch for the accuracy and completeness of the data, and decided to submit the article for publication. The manufacturer of Helixor-M provided study drugs and information for the IND but played no role in the study design, execution, or result interpretation.

### Statistical Analysis

Patient baseline characteristics and AEs were summarized, and the number of patients treated at each dose level and their DLTs were tabulated. AEs were tabulated by type and grade. The best objective response and time to progression were plotted for individual patients. Kaplan–Meier curves were plotted for progression-free survival and OS. A pairwise Wilcoxon signed-rank test was used for FACT-G QoL.

### Data Availability

The trial data are available upon request through communication with the corresponding author.

## Results

### Baseline Patient Characteristics

Between March 2017 and January 2021, 21 patients with advanced solid tumors, including 7 colorectal, 3 ovarian, 2 pancreatic, and 1 each with appendix, basal cell, breast, lung, melanoma, neuroendocrine, salivary, synovial sarcoma, and uterine leiomyosarcoma cancer, were treated with ME. The median follow-up duration was 15.3 weeks (range, 2–101.1 weeks). All patients were included in the efficacy and safety analyses.

The median age was 57 years (range, 34–81 years), and 14 patients (66.7%) were female. ECOG performance status was 0 for 5 patients, 1 for 12 patients, and 2 for 4 patients (Table 1). Among the 21 patients, 20 (95.2%) had received chemotherapy, 5 (23.8%) immunotherapy, 12 (57.1%) targeted therapy, 9 (42.9%) radiotherapy, and 17 (81%) surgery. Sixteen patients (76.2%) received at least two previous systemic therapies.

**TABLE 1:** Baseline characteristics by dose level

	150 mg (*N* = 8)	300 mg (*N* = 3)	600 mg (*N* = 8)	900 mg (*N* = 2)	Overall (*N* = 21)
**Gender**					
Female	7 (87.5%)	2 (66.7%)	4 (50.0%)	1 (50.0%)	14 (66.7%)
Male	1 (12.5%)	1 (33.3%)	4 (50.0%)	1 (50.0%)	7 (33.3%)
**Age**					
Mean (SD)	56.4 (11.3)	57.6 (3.2)	61.9 (11.6)	50.4 (5.4)	58.1 (10.3)
Median (range)	58.0 (34.4–71.1)	56.0 (55.5–61.3)	64.7 (41.9–81.4)	50.4 (46.6–54.2)	57.4 (34.4–81.4)
**Eastern Cooperative Oncology Group**					
0	1 (12.5%)	1 (33.3%)	2 (25.0%)	1 (50.0%)	5 (23.8%)
1	6 (75.0%)	1 (33.3%)	5 (62.5%)	0 (0%)	12 (57.1%)
2	1 (12.5%)	1 (33.3%)	1 (12.5%)	1 (50.0%)	4 (19.0%)
**Race**					
Black/African American	1 (12.5%)	0 (0%)	1 (12.5%)	2 (100%)	4 (19.0%)
White	7 (87.5%)	2 (66.7%)	6 (75.0%)	0 (0%)	15 (71.4%)
Other	0 (0%)	1 (33.3%)	1 (12.5%)	0 (0%)	2 (9.5%)
**Ethnicity**					
Non-Hispanic	8 (100%)	3 (100%)	6 (75.0%)	2 (100%)	19 (90.5%)
Hispanic	0 (0%)	0 (0%)	2 (25%)	0 (0%)	2 (9.6%)
**Prior surgery**					
No	1 (12.5%)	0 (0%)	2 (25.0%)	1 (50.0%)	4 (19.0%)
Yes	7 (87.5%)	3 (100%)	6 (75.0%)	1 (50.0%)	17 (81.0%)
**Prior chemotherapy**					
No	0 (0%)	0 (0%)	1 (12.5%)	0 (0%)	1 (4.8%)
Yes	8 (100%)	3 (100%)	7 (87.5%)	2 (100%)	20 (95.2%)
**Prior radiotherapy**					
No	4 (50.0%)	1 (33.3%)	5 (62.5%)	2 (100%)	12 (57.1%)
Yes	4 (50.0%)	2 (66.7%)	3 (37.5%)	0 (0%)	9 (42.9%)
**Prior targeted therapy**					
No	3 (37.5%)	0 (0%)	5 (62.5%)	1 (50%)	9 (42.9%)
Yes	5 (62.5%)	3 (100%)	3 (37.5%)	1 (50%)	12 (57.1%)
**Prior immunotherapy**					
No	6 (75.0%)	2 (66.7%)	6 (75.0%)	2 (100%)	16 (76.2%)
Yes	2 (25.0%)	1 (33.3%)	2 (25.0%)	0 (0%)	5 (3.8%)
**Prior lines of therapy**					
Mean (SD)	3.4 (3.3)	3.7 (2.1)	2.3 (1.3)	4.5 (3.5)	3.1 (2.4)
Median (range)	2.5 (1.0, 11.0)	3.0 (2.0, 6.0)	2.0 (1.0, 4.0)	4.5 (2.0, 7.0)	2.0 (1.0, 11.0)

Abbreviation: SD: standard deviation.

### Safety

The MTD was determined to be 600 mg three times a week. Table 2 summarizes the number of patients treated at each dose level and the number who experienced DLT. Of the 21 patients, only 3 (14.7%) discontinued treatment because of a DLT: at the 150 mg dose level, a neuroendocrine tumor patient discontinued therapy after three doses of treatment due to grade 3 fatigue. This patient's tumor replaced most of her liver parenchyma and demonstrated tumor necrosis on a CT scan. Her fatigue improved with lactulose, but she was enrolled in hospice. At the 900 mg dose level, a patient with colon cancer discontinued treatment after nine doses because of grade 3 alanine aminotransferase (ALT) elevation that resolved when treatment was discontinued. Also, at 900 mg, a patient with breast cancer discontinued treatment after four doses because of grade 3 flank pain and dyspnea. All four of these AEs were considered by investigators to be related to treatment.

**TABLE 2:** Dose-level summary with DLTs

Dose level	Dose	Mean/median number of doses administered	DLT count (%) and number of patients	DLTs
1	150 mg	22.2/21	1 of 8 (12.5%)	Grade 3 fatigue
2	300 mg	22.7/21	0 of 3 (0.0%)	
3	600 mg	17.9/13.5	0 of 8 (0.0%)	
4	900 mg	6.5/6.5	2 of 2 (100%)	Grade 3 alanine aminotransferase elevation (*N* = 1) Grade 3 dyspnea/ flank pain (*N* = 1)

Note: Four patients, 2 each at dose levels 150 and 600 mg, missed more than 25% of the first 4 weeks of treatments. Therefore, 2 additional patients were recruited for each dose level.

Abbreviations: DLT: dose-limiting toxicity.

AEs of any grade were reported in 20 of 21 patients (95.2%; Table 3 AEs of any grade with a greater than 5% incidence and Supplementary Table S1 AEs of any grade with a 5% or lower incidence). The most common AEs, regardless of causality, were fatigue (52.4%), nausea (33.3%), and limb edema (23.8%). Grade 3 or 4 AEs occurred in 50% of patients. Treatment-related AEs occurred in 13 of 21 patients (61.9%; Supplementary Table S2). The most common treatment-related AEs were fatigue (28.6%), nausea (9.5%), and chills (9.5%). Most treatment-related AEs (76.9%) were grade 1, with grade 3 events noted in 14.8% of patients. No grade 4 or 5 treatment-related AEs were reported.

**TABLE 3:** Summary of all AEs (*N* = 21, any grade incidence greater than 5%)

	Any grade	Grade 1	Grade 2	Grade 3	Grade 4
Any events	20 (95.2%)	2 (15%)	7 (35%)	8 (40%)	2 (10%)
Fatigue	11 (52.4%)	7 (33.3%)	2 (9.5%)	2 (9.5%)	0 (0%)
Abdominal pain	7 (33.3%)	5 (23.8%)	2 (9.5%)	0 (0%)	0 (0%)
Nausea	7 (33.3%)	4 (19%)	3 (14.3%)	0 (0%)	0 (0%)
Edema limbs	5 (23.8%)	3 (14.3%)	2 (9.5%)	0 (0%)	0 (0%)
Pain	5 (23.8%)	3 (14.3%)	2 (9.5%)	0 (0%)	0 (0%)
Diarrhea	4 (19%)	3 (14.3%)	1 (4.8%)	0 (0%)	0 (0%)
Dyspnea	4 (19%)	1 (4.8%)	1 (4.8%)	2 (9.5%)	0 (0%)
Skin and subcutaneous tissue disorders	4 (19%)	3 (14.3%)	1 (4.8%)	0 (0%)	0 (0%)
Anemia	3 (14.3%)	1 (4.8%)	0 (0%)	2 (9.5%)	0 (0%)
Anorexia	3 (14.3%)	2 (9.5%)	1 (4.8%)	0 (0%)	0 (0%)
Chills	3 (14.3%)	3 (14.3%)	0 (0%)	0 (0%)	0 (0%)
Constipation	3 (14.3%)	1 (4.8%)	2 (9.5%)	0 (0%)	0 (0%)
Gastrointestinal disorders	3 (14.3%)	3 (14.3%)	0 (0%)	0 (0%)	0 (0%)
Lung infection	3 (14.3%)	0 (0%)	1 (4.8%)	2 (9.5%)	0 (0%)
Sinus tachycardia	3 (14.3%)	0 (0%)	2 (9.5%)	1 (4.8%)	0 (0%)
Acute kidney injury	2 (9.5%)	0 (0%)	0 (0%)	2 (9.5%)	0 (0%)
Back pain	2 (9.5%)	1 (4.8%)	1 (4.8%)	0 (0%)	0 (0%)
Dizziness	2 (9.5%)	1 (4.8%)	1 (4.8%)	0 (0%)	0 (0%)
Fall	2 (9.5%)	0 (0%)	2 (9.5%)	0 (0%)	0 (0%)
Flank pain	2 (9.5%)	0 (0%)	1 (4.8%)	1 (4.8%)	0 (0%)
Gastroesophageal reflux disease	2 (9.5%)	0 (0%)	2 (9.5%)	0 (0%)	0 (0%)
Generalized muscle weakness	2 (9.5%)	1 (4.8%)	0 (0%)	1 (4.8%)	0 (0%)
Headache	2 (9.5%)	2 (9.5%)	0 (0%)	0 (0%)	0 (0%)
Insomnia	2 (9.5%)	1 (4.8%)	0 (0%)	1 (4.8%)	0 (0%)
Localized edema	2 (9.5%)	2 (9.5%)	0 (0%)	0 (0%)	0 (0%)
Nasal congestion	2 (9.5%)	1 (4.8%)	1 (4.8%)	0 (0%)	0 (0%)
Neoplasm benign-maligant-unspecified (inc cyst-plyp)	2 (9.5%)	0 (0%)	0 (0%)	1 (4.8%)	1 (4.8%)
Peripheral sensory neuropathy	2 (9.5%)	1 (4.8%)	1 (4.8%)	0 (0%)	0 (0%)
Rash acneiform	2 (9.5%)	1 (4.8%)	1 (4.8%)	0 (0%)	0 (0%)

### Reasons for Discontinuation

Fifteen of the 21 patients (71.4%) discontinued because of disease progression (Supplementary Table S3). Nine had RECIST-defined progressive disease. The other 6 were experiencing clinical progression. Three patients (14.3%) withdrew consent (2 patients were had stable disease at month 5 and decided to pursue subcutaneous mistletoe treatment, and 1 had increased fatigue and a desire to be closer to family). The remaining 3 patients discontinued treatment because of DLTs.

### Clinical Activity

**FIGURE 1:**
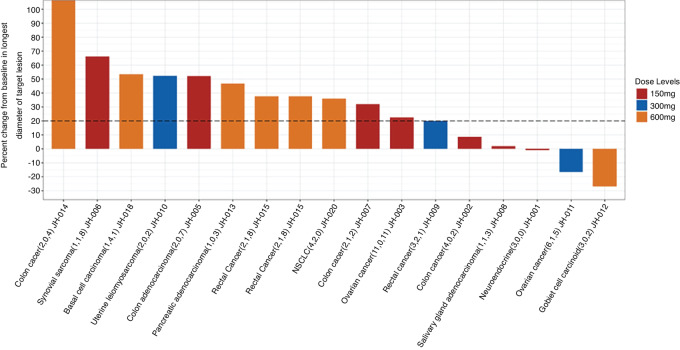
Best objective response. NSCLC: non–small cell lung cancer; PD: progressive disease; SD: stable disease. Note: Waterfall plot of patients treated with Helixor-M and had follow-up scans after baseline to evaluate tumor response (*N* = 17). Their best tumor response is shown as the percentage change from baseline in the longest diameter of the target lesion. The numbers in parentheses are the number of prior systemic therapies (including targeted therapy, hormonal therapy, immunotherapy, or chemotherapy), the number of prior radiation treatments, and the number of surgeries.

Seventeen patients (80.9%) had follow-up scans after baseline to evaluate tumor response. Their best tumor response is shown in Fig. 1. Objective responses were not observed. Stable disease was observed in 5 patients: 1 each with a neuroendocrine tumor, ovarian cancer, colon cancer, goblet cell carcinoid, and salivary gland adenocarcinoma. Prior to enrolling in the trial, the neuroendocrine tumor patient had disease progression to her liver after three lines of therapy; the patient with ovarian cancer had rapidly progressive disease after six lines of treatment; the patient with colon cancer had metastatic disease progressing to her lungs with rapidly rising CEA after four lines of therapy; the patient with goblet cell carcinoid had rapidly progressing disease requiring surgical debulking after three lines of treatment; and the salivary gland patient had metastatic disease in her lung after discontinuing an experimental FGFR inhibitor due to toxicity. Among patients with stable disease, 3 (out of 7) were at a dose of 150 mg, 1 (out of 3) was at 300 mg, and 1 (out of 8) was at 600 mg. Three patients experienced reductions in baseline target lesions: a patient with neuroendocrine cancer at 150 mg, a patient with ovarian cancer at 300 mg, and a patient with appendix goblet cell cancer at 600 mg (Fig. 1). They had received three, six, and three lines of prior systemic therapy, respectively.

**FIGURE 2:**
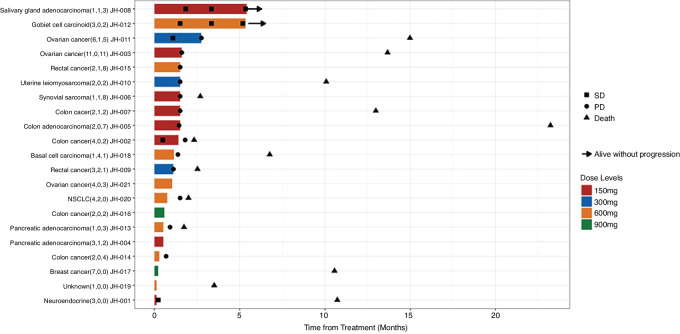
Time to stable disease, progressive disease, and death. NSCLC: non–small cell lung cancer; PD: progressive disease; SD: stable disease. Note: Time to stable disease, progression, and death of all patients treated with Helixor-M (*N* = 21). The treatment duration is shown as the length of bars. The numbers in the parentheses are the number of prior systemic therapies (including targeted therapy, hormonal therapy, immunotherapy, or chemotherapy), the number of prior radiation treatments, and the number of surgeries. The time to death should be interpreted with caution, as patients received different subsequent treatments.

Figure 2 shows the treatment duration, time with stable disease, time to progressive disease, and time to death. Fourteen of the 21 patients (66.7%) had died at the time of the analysis, and of those, 11 had progressive disease before death. In 3 (JHU-001, 005, 013) of the 5 patients who had stable disease, a patient with appendix goblet cell carcinoid and a patient with salivary gland adenocarcinoma experienced stable disease for more than 5 months, and an ovarian patient experienced stable disease for 2.5 months. The median time from stable disease to progression/death/last follow-up was 15 weeks (range: 5.7–45.7 weeks).

### Tumor Marker Kinetics

Serum CEA was positive in 7 patients (JHU-002, 009, 005, 007, 014, 015, 016) and increased over time in 3 patients at doses of 150 and 300 mg, and was stable over time in 2 patients with doses of 600 and 900 mg; the remaining 2 patients had baseline CEA level only (Supplementary Fig. S1).

**FIGURE 3:**
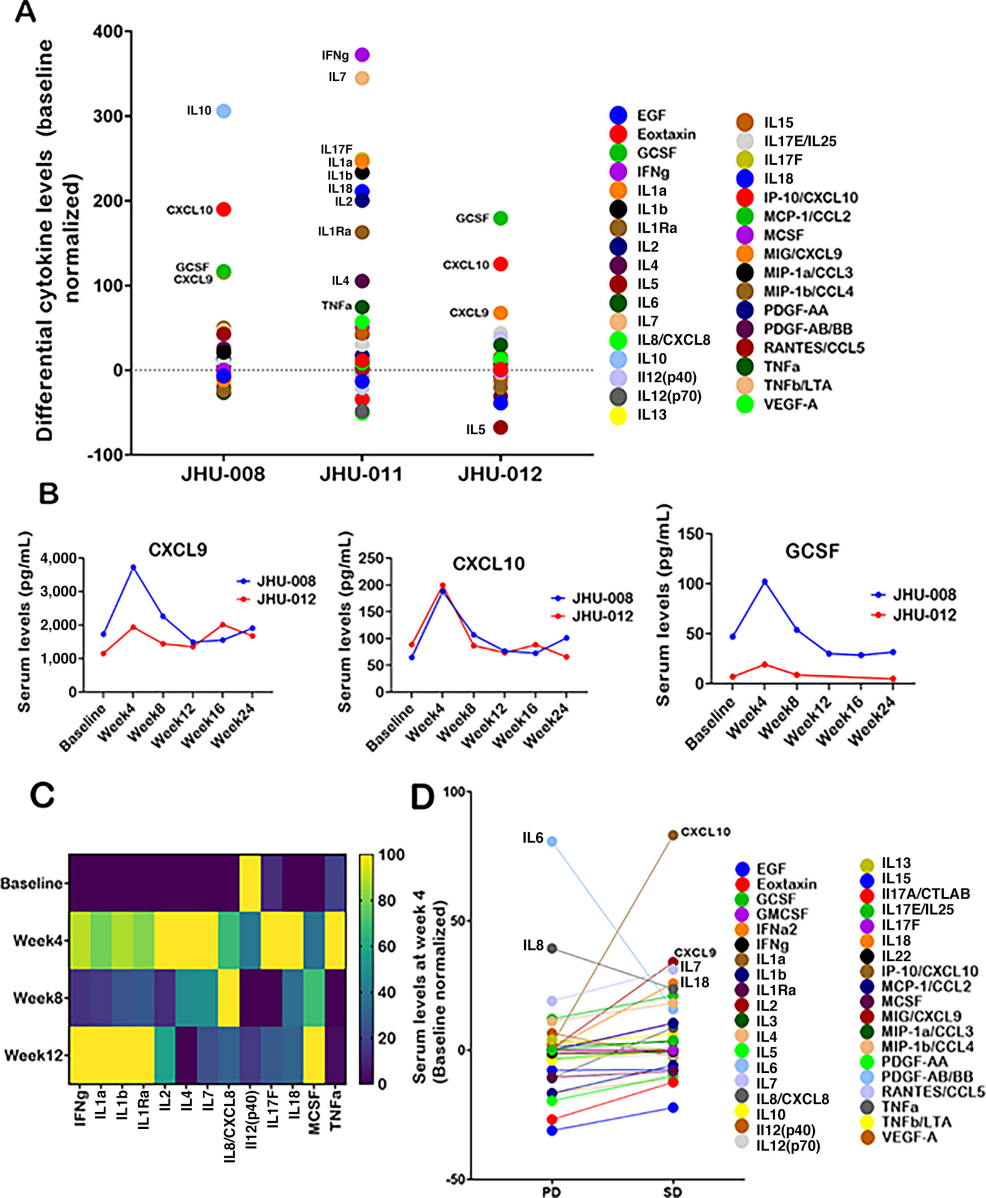
Chemokines, cytokines, and growth factors in patients showing stable disease. Note: Serum cytokine/chemokine/growth factor profiling. **A,** Superimposed scatter plot showing levels of cytokines, chemokines, and growth factors in patients JHU-008, JHU-011, and JHU-012 at week 4. Analytes showing greater changes (up or down) as compared with baseline are labeled in the plot and were selected for multi-timepoint analysis. **B,** Line graph showing levels (pg/mL) of CXCL10, CXCL9, and GCSF in serum samples of patients JHU-008 and JHU-012. **C,** Heatmap showing serum levels (pg/mL) of selected analytes from scatter plot for patient JHU-011. **D,** Superimposed scatter plot showing median levels of cytokines, chemokines, and growth factors in patients group showing either progressive or stable disease. Slope of the line indicate if a particular analyte was higher or lower in the stable disease group as compared with patients with progressive disease. Factors that show up or downregulation in the stable disease group are labeled in the plot. IL3 was removed from the temporal analysis because it was under the lower detection limit in all the subjects. IFNα2 was also removed from the investigation because it was either not detectable or unchanged compared with baseline levels.

Serum cytokines, chemokines and growth factors were analyzed. The percent change for all the analytes was calculated by normalizing them to respective baseline levels. Supplementary Figure S2 shows normalized differential expression levels for all analytes at week 4. Of 13 patients, 7 (JHU-006, -008, -010, -011,-012, -013, and -018) showed elevated IL1a, IL1b, IL7, IL10, IL12, IL17F, IL18, and IFNγ. Among the subset, JHU-008, -011, and -012 showed stable disease and were analyzed separately to identify differential cytokine levels (Fig. 3A). Patients JHU-008 and JHU-012 had a similar trend for CXCL10, CXCL9, and GCSF, which peaked at week 4 (Fig. 3B). Furthermore, IL10 was found to increase only in JHU-008. Patient JHU-011 showed the most activated immune profile with elevated IFNγ, IL7, IL17F, IL1a, IL1b, IL18, IL2, and TNFα at week 4 (Fig. 3C). Patients with stable disease showed increased CXCL10, CXCL9, IL7, and IL8, while others demonstrated higher IL6 and IL8 (Fig. 3D).

### Longitudinal Outcomes and QoL

The median progression-free survival was 46 days [95% confidence interval (CI), 44–48 days]. The median OS was 10.1 months (95% CI, 3.5 months to not reached). No significant difference was observed across dose levels. The median total score of FACT-G first increased from 79.7 [interquartile range (IQR), 65.5–93] at week 1 to 93 (IQR, 88–100) at week 4 and then decreased slightly to 89 (IQR, 78.2–98) at the end of treatment. Similar trends were observed in physical, social/family, emotional, and functional wellbeing. Though statistically nonsignificant, patients’ QoL measured by FACT-G improved by the end of treatment from week 1 in the total score and in physical, social/family, and functional well-being, with the improvement greater in patients who experienced stable disease than in patients whose disease progressed without stabilizing (Supplementary Fig. S3–S12).

## Discussion

To our knowledge, this was the first phase I trial to assess the safety and antitumor activity of intravenous Helixor-M in patients with heavily pretreated advanced solid tumors. We found that the MTD was 600 mg three times a week. The product was found to have an overall manageable safety profile. One patient discontinued treatment within 600 mg, the MTD level, due to treatment-related fatigue. The most common treatment-related AEs were fatigue, nausea, and chills. Grade 3 or 4 treatment-related AEs occurred in 14.8% of patients. Although 5 patients had stable disease and 3 patients experienced tumor reduction receiving a dose within the MTD, there were no objective responses. Serum CEA levels that were positive at baseline stabilized for patients at higher doses, suggesting a dose–response effect. Though statistically nonsignificant, patients’ QoL improved by the end of treatment in total score and in physical, social/family, and functional wellbeing.

The safety profile of Helixor-M observed in this study is similar to that of Helixor-P reported in Huber and colleagues (23). In both trials, 21 patients with advanced cancer were treated with MEs. While this study required patients to have had disease progression after at least one systemic therapy, Huber and colleagues did not. The Huber and colleagues trial reported treatment-related AEs including fever (*N* = 4), weakness (*N* = 3), eosinophilia (*N* = 2), and temporary elevation of ALT (*N* = 2). We observed similar treatment-related AEs: chills (*N* = 4), fatigue (*N* = 6), and temporary ALT elevation (*N* = 1). On weekly administration, the MTD was not reached for Helixor-P at a dose level of 2,000 mg in Huber and colleagues. Given at a higher frequency (three times a week), the MTD was 600 mg for Helixor-M in this study. Comparing the two studies, patients in this study were more heavily pretreated with chemotherapy(85.7% vs. 66.7%) and immunotherapy (28.6% vs. 19%), possibly contributing to a lower MTD due to lower tolerability. Temporary improvement of tumor markers CA19-9 and calcitonin and stable disease followed by slow progression were observed in patients receiving Helixor P.

Serum cytokine profiles varied markedly among the subjects due to tumor type differences. Patients with stable disease showed elevated CXCL9 and CXCL10. CXCL9, CXCL10, and CXCL11 share a common receptor, CXCR3, and can mediate the recruitment of cytotoxic T and natural killer cells to solid tumors (25). Higher levels of CXCL9, CXCL10, and GCSF posttreatment indicate possible immune activation (26). The serum profile of patient JHU-011 showed a dramatic increase in cytokines, indicating immune activation. Noteworthy among these are IFNγ, which plays a role in both innate and adaptive antitumor response, and IL7, which presents antitumor effects by increasing CD8^+^ T-cell infiltration, and is also the subject of cancer trials (27, 28). Overall, these cytokines indicate antitumor serum profiles in patients with stable disease (29). Furthermore, patients without stable disease had a larger increase in IL6 and IL8. IL6 can promote tumor-cell proliferation, survival, invasiveness, and metastasis (30), while IL8 correlates with an immunosuppressive tumor microenvironment resulting in adverse cancer prognosis (31). However, the mere presence of these cytokines should not be considered a direct activity of ME, and mechanistic studies are highly warranted. Pharmacokinetic data were not collected in this study. As Helixor M is a ME formulation with multiple bioactive compounds, developing a bioanalytical method to analyze the plasma/serum levels is challenging. To date, no methods certified by Clinical Laboratory Improvement Amendments are available.

Systematic reviews summarized research findings regarding the effects of ME on QoL, chemotherapy toxicity, survival, cancer-related fatigue, and safety. A systematic review suggests that positive effects of ME on QoL and chemotherapy toxicity were found in most RCTs. However, these RCTs had methodologic deficits (e.g., a lack of blinding, high attrition; ref. 32). And most of these RCTs show low-grade AEs but no survival benefit associated with ME (32). Another systematic review found a significant improvement in QoL with ME treatment. That review confirmed the robustness of results by sensitivity analyses against methodologic and study design moderators (33). Similar QoL benefits of ME were reported in other systematic reviews, one of which found that ME has a moderate effect on cancer-related fatigue similar to that seen with physical activity (34, 35).

In conclusion, ME has common toxicities with manageable safety profiles. The MTD is likely dependent upon dose frequency and disease severity. Although no objective responses were recorded in this study, stable disease and tumor shrinkage were observed in some heavily pretreated patients (one to six lines of prior therapy). Moreover, a trend toward stabilization of tumor markers at higher doses was observed, suggesting that higher doses may have led to better responses had less severely ill patients been treated. Finally, ME may improve the QoL of patients with heavily pretreated advanced solid tumors. Improved QoL may enable patients to tolerate therapy longer. Future phase II trials can explore the optimal timing and dose frequency of intravenous mistletoe extracts. Furthermore, interested investigators can combine ME infusions with chemotherapy or targeted therapy and assess the impact of the infusions on treatment time and patient QoL along with functional immune correlates with rigorously designed and conducted RCTs.

## Supplementary Material

Supplementary Materials and Methods SM1 Supplementary Materials and Methods show the trial protocol

Table ST1 Table S1 shows summary of all adverse events

Table ST2 Table S2 shows treatment-related adverse events by dose level

Table ST3 Table S3 shows reasons for discontinuation with patient charateristics

Figure S1 Figure S1 shows tumor marker over time

Figure S2 Figure S2 shows summary of normalized serum levels of cytokins/chemokines/growth factors at week 4

Figure S3 Figure S3 shows functional assessment of cancer therapy - general over time

Figure S4 Figure S4 shows functional assessment of cancer therapy - general over time by response

Figure S5 Figure S5 shows physical wellbeing over time

Figure S6 Figure S6 shows physical well-being over time by response

Figure S7 Figure S7 shows social/family well-being over time

Figure S8 Figure S8 shows social/family well-being over time by response

Figure S9 Figure S9 shows emotional well-being over time

Figure S10 Figure S10 shows emotional well-being over time by response

Figure S11 Figure S11 shows functional well-being over time by response

Figure S12 Figure S12 shows functional well-being over time by response
