# Supplementary material for: Phase I Trial of Intravenous Mistletoe Extract in Advanced Cancer
Source: Cancer Res Commun. 2023 Feb 28;3(2):338–46. doi: 10.1158/2767-9764.CRC-23-0002 (PMC9973409; doi:10.1158/2767-9764.CRC-23-0002)
Supplement: Supplementary Materials and Methods SM1 — Supplementary Materials and Methods show the trial protocol [file crc-23-0002-s01.pdf]

TITLE: A Phase I Dose Escalating Trial of Mistletoe Extract in Patients with Advanced Solid Tumors

Protocol #: J1681, IRB00090991

Principal Investigator: Channing Paller, MD (Protocol Chair)
The Sidney Kimmel Comprehensive Cancer Center
201 N. Broadway, Viragh 9125
Baltimore, MD 21287 Phone: 410-955-8239,
Fax: 410-614-8397, Email: (cpaller1@jhmi.edu)

Statistician:
Hao Wang, Ph.D.
550 N Broadway Suite 1101
Baltimore, MD 21205
410-614-3426/Fax 410-955-0859
hwang76@jhmi.edu

Brandon Luber, Sc.M.
550 N Broadway Suite 1101
Baltimore, MD 21205
bluber1@jhmi.edu

Study Coordinator:
Christina Raynor
201 N. Broadway, Viragh 9th Floor
Baltimore, MD 21287
410-955-5096/Fax 410-955-5097
craynor1@jhmi.edu

Lead Research Nurse:
Pat Fischer
201 N. Broadway, Viragh 9th Floor
Baltimore, MD 21287
410-502-0662/Fax 410-955-5097
pfische6@jhmi.edu

Molly Geare
201 N. Broadway, Viragh 9th Floor
Baltimore, MD 21287
410-955-6610/Fax 410-955-5097

Regulatory Specialist:
Jennifer Durham, PhD
1650 Orleans St, CRB I Room 489
Baltimore, MD 21287
410-502-3038/Fax 410-614-8216
juram1@jhmi.edu

IND: IND 128726

IND Sponsor: Channing Paller, M.D.
201 N. Broadway, Viragh 9125
Baltimore, MD 21287
410-955-8239/ 410-955-5097(f)
cpaller1@jhmi.edu

Investigational Agent: Helixor® M (Mistletoe Extract), Helixor Heilmittel GmbH & Co. KG

**Protocol Type / Version # / Version Date:**	Original, Version 1.0, November 15, 2015<br>Original, Version 1.1, December 8, 2015<br>Original, Version 1.2, December 21, 2015<br>Original, Version 1.3, April 3, 2016<br>Original, Version 1.4, July 20, 2016<br>Amendment 1, Version 2.0, February 13, 2017<br>Amendment 2, Version 3.0, August 24, 2017<br>Amendment 3, Version 4.0, March 1, 2018<br>Amendment 4, Version 5.0, April 23, 2018<br>Amendment 5, Version 6.0, January 14, 2019<br>Amendment 6, Version 7.0, April 11, 2019<br>Amendment 7, Version 8.0, July 7, 2019

TABLE OF CONTENTS

Page

1. OBJECTIVES 5
   1.1 Primary Objectives 5
   1.2 Secondary Objectives 5
   1.3 Exploratory Objectives 5
   1.4 Study Design 5

2. BACKGROUND 6
   2.1 Helixor® M 7
   2.2 Rationale 8

3. ELIGIBILITY CRITERIA 8
   3.1 Inclusion Criteria 8
   3.2 Exclusion Criteria 9
   3.3 Inclusion of Women and Minorities 11

4. REGISTRATION PROCEDURES 11
   4.1 General Guidelines 11
   4.2 Registration Process 11

5. TREATMENT PLAN 11
   5.1 Agent Administration 11
   5.2 Dosing Criteria 13
   5.3 Contraception, Use in Pregnancy, Use in Nursing 13
   5.4 Definition of Dose-Limiting Toxicity (DLT) and Maximum Tolerated Dose (MTD) 14
   5.5 General Concomitant Medication and Supportive Care Guidelines 15
   5.6 Duration of Therapy 17
   5.7 Duration of Follow Up 18
   5.8 Criteria for Removal from Study 18

6. DOSING DELAYS AND SCHEDULING/DOSE MODIFICATIONS 19
   6.1 Dosing Delays and Scheduling/Missed Doses 19
   6.2 Dose Modifications 19

7. ADVERSE EVENTS: LIST AND REPORTING REQUIREMENTS 19
   7.1 Definitions 20
   7.2 Relationship 21
   7.3 Expectedness 21
   7.4 Handling of Expedited Safety Reports 21
   7.5 Reporting 22
   7.6 Possible Adverse Events 23

8. PHARMACEUTICAL INFORMATION 23

8.1 Mode of Action 23
8.2 Product description 23
8.3 Storage and Labeling 24
8.4 Stability 24
8.5 Preparation and Administration 24
8.6 Patient Care Implications 25
8.7 Returns and Reconciliation 25

9. CORRELATIVE/SPECIAL STUDIES 25
9.1 Peripheral Blood Mononuclear Cells (PBMC) Studies 25
9.2 Serum Studies 25
9.3 Plasma Studies 26

10. STUDY CALENDAR 27

11. MEASUREMENT OF EFFECT 30

12. DATA REPORTING / REGULATORY REQUIREMENTS 31
12.1 Data Management 32
12.2 Safety Meetings 33
12.3 Monitoring 33

13. STATISTICAL CONSIDERATIONS 33
13.1 Study Design/Endpoints 33
13.2 Sample Size/Accrual Rate 35
13.3 Analysis of Primary/Secondary Endpoints 35
13.4 Reporting and Exclusions 36

REFERENCES 38

APPENDIX A: Performance Status Criteria 40

APPENDIX B: SAE Reporting Form 41

APPENDIX C: FACT-G Questionnaire (Version 4) 44

APPENDIX D: Response Evaluation Criteria in Solid Tumors (RECIST) 1.1 Criteria for Evaluating Response in Solid Tumors 46

APPENDIX E: Immune Related Response Criteria (irRC) 49

1. OBJECTIVES

1.1 Primary Objectives

1.1.1 To assess the safety and characterize the toxicity of Helixor® M mistletoe extract administered intravenous (IV) in patients with advanced solid tumors.

1.1.2 Identify the maximum tolerated dose (MTD)

1.2 Secondary Objectives

1.2.1 To measure tumor marker kinetics in patients receiving treatment.

1.3 Exploratory Objectives

1.3.1 To estimate progression-free survival (PFS) and time to progression (TTP) in patients treated with Helixor® M mistletoe extract using RECIST 1.1 and irRC.

1.3.2 To collect peripheral blood mononuclear cells (PBMCs), serum, and plasma to explore potential therapeutic targets, biomarkers, and predictors of treatment response (PFS and best overall response).

1.3.3 To assess tumor burden dynamics using exploratory circulating biomarkers in serial collections of sera and plasma at baseline and throughout treatment.

1.3.4 To collect pre and post-treatment whole blood to evaluate germline mutations in circulating DNA that may correlate with clinical benefit (PFS and best overall response).

1.3.5 To determine changes in cytokine production by peripheral blood mononuclear cells (PBMC) as indicated by interleukin 2 [IL-2], interleukin 6 [IL-6], interleukin 8 [IL-8], interleukin 12 [IL-12], and interferon- gamma [IFN-γ].

1.4 Study Design

This study is a multi-center Phase I study that consists of two phases: the dose escalation phase and an expansion phase. The dose escalation phase will be used to evaluate the safety and toxicity of Helixor® M (mistletoe extract). An accelerated titration design (ATD) will be utilized to determine the MTD. The expansion phase will be used to obtain preliminary efficacy data about Helixor® M.

This phase I study will evaluate up to 8 dose levels of Helixor® M as a single agent: 150 mg, 300 mg, 600 mg, 900 mg, 1200 mg, 1500 mg, 1800 mg, and 2000 mg. A lead in dose of 50 mg of Helixor® M will be administered to each patient for their first dose. Rationale for a maximum dose of 2000 mg was determined through personal correspondence with Dr. Roman Huber regarding his ongoing clinical trial with IV Helixor® P (Helixor® Infusion Study: a prospective dose finding study. Study Protocol Phase I, HIS-I-730-127-2012, EudraCT-no.: 2012-004189-16). Patients will be assigned to a dose level in the order

of study entry. The first cohort of 1 subject will receive mistletoe at the 150 mg dose level. One subject will be enrolled in each cohort until a subject experiences a drug related toxicity of grade 2 or higher, whereupon an additional 2 patients will be enrolled at that dose level. Thereafter, the study will become the traditional 3+3 design with 3 or 6 patients treated at this dose level and at all subsequent dose levels depending upon the incidence of a Dose Limiting Toxicity (DLT). If MTDs are not determined at the dose of 2000 mg, this will be the dose taken to expansion phase of the trial and no further escalation of dose is planned.

A subject who is withdrawn from the study prior to completion of the DLT period for a reason other than a DLT and has not experienced a study drug-related adverse event of ≥ grade 2 may be replaced.

2. BACKGROUND

The treatment of cancer with mistletoe extracts were first introduced by Rudolf Steiner in the 1920s. However, the use of mistletoe for its medicinal properties to treat numerous human ailments dates back several thousand years[1]. Commercially available mistletoe extracts are prepared from *Viscum album* Linnaeus (*Viscum album* L. or European mistletoe). *Viscum album* L. is a semi-parasitic plant that grows on several different types of trees. The extracts derived from mistletoe contain numerous ingredients in varying concentrations depending on the species of the host tree (e.g. apple, elm, oak, pine, birch, poplar, maple, and spruce), the time of year harvested (summer versus winter), and the pharmaceutical extract processing procedures (fermented or unfermented). Commercially available extracts of mistletoe in Europe include ABNOBAviscum®, Cefalektin®, Eurixor®, Helixor®, Iscador®, Iscucin®, Isorel® and Lektinol®[2]. Mistletoe extracts are one of the most frequently prescribed therapies for cancer in Europe, especially in Germany, Switzerland, and Austria[2].

Several biologically active substances are found in mistletoe extracts: lectins, viscotoxins, peptides, amino acids, flavonoides, oligo and polysaccharides, and other substances in low concentrations[2]. Mistletoe extract has two mechanisms of action, tumor cytotoxicity and immunomodulation. The cytotoxic effects of mistletoe extract are a result of protein synthesis interference[3,4], cell cycle inhibition[5], and inducing apoptosis[5-8]. The immunomodulatory properties of mistletoe extract work through increasing white blood cell numbers[1], as well as stimulating the production of immune enhancing cytokines, such as IL-1, IL-6, IFNγ, TNFα[9,10]. Lastly, mistletoe extract has been shown to have anti-angiogenic properties[11,12].

Mistletoe lectins are the most widely studied component of mistletoe extract. Four different lectins have been identified: ML-1, ML-2, ML-3, and *Viscum album* chitin-binding agglutinin. Lectins are composed of two subunits, α and β, that when transported into the cell via endocytosis are dissociated from one another and can mediate different functions. The α subunit has been shown to interfere with protein synthesis through ribosome-inactivation, therefore inducing apoptosis, while the sugar binding β subunit has been shown to have more immunomodulating characteristics, such as NK cell, lymphocyte, and macrophage activation[13-15]. Similar to mistletoe lectins, viscotoxins, peptides, oligo and polysaccharides, and flavanoids have duel cytotoxic and immunostimulatory actions[1].

2.1 Helixor® M

Helixor® products are derived from three subspecies of white-berry mistletoe growing on different host trees: Helixor® A from fir mistletoe (abietis), Helixor® M from apple tree mistletoe (mali), and Helixor® P from pine mistletoe (pini). Helixor® M preparations have been recommended for lower abdomen (bladder, uterus, and ovaries), abdomen (stomach, colon, liver, pancreas), and breast cancers. Helixor® A preparations have been primarily used to treat head and neck cancers (including brain tumors), lung cancer, and prostate cancers, whereas Helixor® P preparations are recommended for malignant melanoma, sarcomas, kidney cancer, testicular cancer, and breast cancers[16].

Helixor® M was registered first in Germany in 1976 and got a marketing authorization according to the new German drug law in 1982. Besides Germany it has marketing authorization in Austria, Canada, Korea, Latvia, Lithuania, Luxemburg, Macedonia, Peru, Russia, Sweden and Switzerland. While mistletoe extracts are not approved for the treatment of cancer patients in the United States, V*iscum album* is listed in the United States Homeopathic Pharmacopoeia. It is estimated that Helixor® has been administered to over 400,000 patients since receiving marketing authorization.

Mistletoe products are currently registered exclusively for subcutaneous (SC) injection and are administered SC three times or twice a week or even daily. Other modes of administration like intracavitary instillation, intralesional injection or high-dose intravenous (IV) infusion are not yet registered, but are often used in clinics or hospitals for palliation in desperate cases after informed consent. There are 27 prospective or retrospective clinical trials investigating Helixor® in a total of 5,809 patients (colorectal, breast, lung and stomach cancer patients as well as patients with liver metastasis, malignant pleural effusions, chronic myeloid leukemia, malignant lymphoma and other cancer types). Systematic reviews confirm improvements in quality of life, tolerability of conventional tumor therapy, survival and/or tumor response[2,17-19].

Helixor® has been administered by intravenous infusion in some special indications since 1975. The main indication for Helixor® intravenous infusion therapy is reduced general condition as well as cancer pain in metastatic disease. The response to infusion therapy in this indication is much better than to standard subcutaneous treatment[20]. In addition, patients with rapid deterioration of their quality of life may profit from this treatment[20]. Tumor regression on high-dose infusion therapy has been demonstrated in some cases showing progressive disease in spite of subcutaneous Helixor® treatment[21]. Among 33 cancer patients treated with high-dose Helixor® infusion, cancer response could be achieved in 9%, no-change in 36%, and a retardation of tumor progression in 27%, going along with a preserved quality of life for at least 6 months[22]. Another study involving 60 cancer patients revealed a decline in ESR and tumor markers in 23% and 17%, respectively, which can be considered to be objective signs of clinical improvement[23]. Given immediately before and during high-dose 5-FU chemotherapy, Helixor® infusion may result in an excellent tolerance of chemotherapy and in an improved quality of life[24]. The clinical experience is largely based on treatment performed at Herdecke Community Hospital and Ludwig-Boltzmann-Institute for Clinical Oncology in Vienna[25,26], as well as the Havelhöhe Community Hospital in Berlin[24]. A prospective clinical trial disclosed no evidence of acute or chronic toxicity in association with high-dose infusions of Helixor®[25].

Review of adverse drug reactions (ADRs) demonstrate that the IV infusion of Helixor® A or M to be safe with the most common reported symptoms to be allergic/pseudoallergic reactions

(generalized itching, tingling sensation, urticaria or exanthema, bronchospasm and Quincke's edema), as well as fever, chills, flu-like symptoms, fatigue, headache and infusion site inflammation. In an observational study recently published comparing ADRs associated with SC versus IV administration of various mistletoe extracts (including Helixor®), it was shown that the incidence of ADRs in patients that received IV mistletoe (4.6%) was lower than those receiving SC mistletoe (8.4%)[27].

2.2 Rationale

Toxicities associated with radiation and chemotherapy diminish quality of life for patients with advanced cancer. Nausea and vomiting, fatigue, mucositis, alteration in memory, and changes in appetite are common side effects with chemotherapy while, radiation can cause fatigue and skin changes. Systematic reviews of subcutaneous Helixor clinical studies confirm improvements in length and quality of life, tolerability of conventional tumor therapy, survival and/or tumor response[2,17-19]. However, clinical studies on the efficacy of high-dose IV infusion of mistletoe products are not yet available. Until now, only a non-GCP compliant safety study on Helixor® IV infusion has been published preceded by a retrospective study Medical experience in the off-label use of Helixor® IV infusion. In the proposed phase I trial, we will seek to determine the safety and toxicity profile as well as the maximum tolerated dose of Helixor® M in patients with advanced solid tumors who have received at least one line of systemic therapy for metastatic disease. We anticipate this trial to be followed by disease-specific phase II trials in colon, pancreas, breast, bladder and/or any solid tumor subtypes where we see a clinical response.

3. ELIGIBILITY CRITERIA

3.1 Inclusion Criteria

3.1.1 Patients with advanced solid tumors and have received first line standard systemic therapy with chemotherapy, immunotherapy, hormonal therapy or other standard treatments for metastatic disease. Patients must either have progressed, are refractory, have stable disease and/or removed from therapy due to toxicities. Patients beyond first line therapy that do not meet criteria may be considered on a case by case basis and allowed at discretion of PI.

3.1.2 Patients with the presence of at least one measurable lesion as defined by RECIST 1.1 criteria for response assessment.

3.1.3 Age $\geq$18 years.

3.1.4 ECOG performance status 0-2 (**Appendix A**).

3.1.5 Life expectancy of greater than 3 months.

3.1.6 Patients must have normal organ and marrow function as defined below (without growth factor or transfusion support within 14 days prior to first dose of investigational product):

– WBC	≥3,000/mcL
– Absolute neutrophil count	≥1,000/mcL
– Platelets	≥90,000/mcL
– Hemoglobin	≥9.0 g/dL
– Total bilirubin	≤1.5 X ULN (patients with diagnosed Gilbert's Syndrome will not be excluded if their direct bilirubin is within normal institutional limits)
– AST(SGOT)/ALT(SGPT)	≤2.5 X ULN
– Creatinine	≤1.5 x ULN OR creatinine clearance ≥50 mL/min/1.73 m^2

3.1.7 Female patient of childbearing potential has a negative urine or serum pregnancy test. If the urine test is positive or cannot be confirmed as negative, a serum pregnancy test will be required. The serum pregnancy test must be negative for the patient to be eligible.

3.1.8 Female patients enrolled in the study, who are not free from menses for >2 years, post hysterectomy / oophorectomy, or surgically sterilized, must be willing to use either 2 adequate barrier methods *or* a barrier method plus a hormonal method of contraception to prevent pregnancy or to abstain from heterosexual activity throughout the study, starting with Visit 1 through 28 days after the last dose of study therapy. Approved contraceptive methods include for example; intra uterine device, diaphragm with spermicide, cervical cap with spermicide, male condoms, female condoms with spermicide, or oral contraceptives. Spermicides alone are not an acceptable method of contraception.

Male patients must agree to use an adequate method of contraception starting with the first dose of study drug through 28 days after the last dose of study therapy.

3.1.9 Ability to understand and the willingness to sign a written informed consent document.

3.2 Exclusion Criteria

3.2.1 Patient with a known history or evidence of brain metastases.

3.2.2 Patients who have had chemotherapy, radiation, hormonal, or biological cancer therapy within 28 days prior to the first dose of study drug excluding patients on long term hormonal therapies who have been on a stable dose for at least 3 months.

3.2.3 Patient is currently participating or has participated in a study of an investigational agent or using an investigational device within 28 days of the first dose of study drug.

3.2.4 Patients who have had surgery within 28 days of dosing of investigational agent, excluding minor procedures (dental work, skin biopsy, etc), celiac plexus block, and biliary stent placement.

3.2.5 Patient is expected to require any other form of systemic or localized antineoplastic therapy while on study.

3.2.6 Patient who has had prior treatment with Mistletoe.

3.2.7 Patients who have received systemic corticosteroids within 28 days prior to the first dose of study drug. Note: Systemic steroid therapy is allowed for subjects on replacement therapy as long as prednisone ≤ 10 mg or its steroid equivalent.

3.2.8 Patients who have received systemic NSAID therapy within 14 days prior to the first dose of study drug.

3.2.9 History of allergic reactions attributed to compounds of similar chemical or biologic composition to Mistletoe.

3.2.10 Uncontrolled intercurrent illness including, but not limited to, ongoing or active infection, symptomatic congestive heart failure, unstable angina pectoris, cardiac arrhythmia, or psychiatric illness/social situations that would limit compliance with study requirements.

3.2.11 Presence of ≥ CTCAE grade 2 toxicity (except peripheral neuropathy and ototoxicity, which are excluded if ≥ CTCAE grade 3) due to prior cancer therapy.

3.2.12 Autoimmune disease: Patients with a history of inflammatory bowel disease, including ulcerative colitis and Crohn's Disease, are excluded from this study, as are patients with a history of symptomatic disease (*e.g.*, rheumatoid arthritis, systemic progressive sclerosis [scleroderma], systemic lupus erythematosus, autoimmune vasculitis [*e.g.*, Wegener's Granulomatosis]); CNS or motor neuropathy considered of autoimmune origin (*e.g.*, Guillain-Barre Syndrome and Myasthenia Gravis, multiple sclerosis). History of Grave's disease on stable thyroid hormone replacement for at least 1 year is allowed.

3.2.13 Patients with a known history of HIV, hepatitis B, hepatitis C, or tuberculosis infection. Patients with a history of cleared hepatitis C (undetectable viral loads) are allowed.

3.2.14 Women with a positive pregnancy test on enrollment or prior to investigational product administration

3.2.15 Women who are pregnant or breastfeeding.

3.2.16 Sexually active fertile men not using effective birth control if their partners are of child bearing potential.

3.2.17 Patient is unwilling or unable to comply with study procedures.

3.3 Inclusion of Women and Minorities

Both men and women of all races and ethnic groups are eligible for this trial.

4. REGISTRATION PROCEDURES

4.1 General Guidelines

Eligible patients will be entered on study centrally at the Sidney Kimmel Comprehensive Cancer Center at the Johns Hopkins University by the Lead Study Coordinator. All sites should contact the Lead Study Coordinator at craynor1@jhmi.edu to verify ongoing study enrollment. The Registration Form and Eligibility Worksheet will be supplied to each participating site.

If a patient does not receive protocol therapy following registration, the patient's registration on the study may be canceled. The Study Coordinator should be notified of cancellations as soon as possible.

4.2 Registration Process

To register a patient, the following documents should be completed and sent to the Protocol Chair and Lead Study Coordinator at the Coordinating Center at craynor1@jhmi.edu:

- Registration Form
- Signed patient consent/HIPAA authorization form
- Eligibility Screening Checklist
- Copy of required screening tests and scans

To complete the registration process, the Coordinating Center Lead Study Coordinator will:

- Assign a patient study number
- Register the patient on the study
- E-mail the Registration Form back to the participating site

5. TREATMENT PLAN

5.1 Agent Administration

Treatment will be administered on an outpatient basis. Reported adverse events and potential risks are described in **Section 7**. Appropriate dose modifications are described in **Section 6**. No investigational or commercial agents or therapies other than those described

below may be administered with the intent to treat the patient's malignancy.

REGIMEN DESCRIPTION				
Agent	***Premedications; Precautions***	***Dose***	***Route***	***Schedule***
**Helixor® M**	No prophylactic pre-medication will be given unless indicated by previous experience in an individual patient per **Section 5.5**.	Per assigned dose level	IV over 3 hours*	M, W, F

* Infusion times are approximate (+/- 15min) and may need to be adjusted based on patient tolerability

**Dose Level**	**# Patient**	**Dose of Helixor® M (mg)**					
		**Week 1**			**Week 2 and beyond**		
		**M**	**W**	**F**	**M**	**W**	**F**
1	1-6	50	150	150	150	150	150
2	1-6	50	300	300	300	300	300
3	1-6	50	600	600	600	600	600
4	1-6	50	900	900	900	900	900
5	1-6	50	1200	1200	1200	1200	1200
6	1-6	50	1500	1500	1500	1500	1500
7	1-6	50	1800	1800	1800	1800	1800
8	1-6	50	2000	2000	2000	2000	2000

Helixor® M will be administered intravenously according to the schedule shown above. The patient must be observed in the clinic for at least 30 minutes after the lead in dose and the first dose of the assigned dose level. Vital signs will be collected prior to each dose. Acute reactions will be managed using guidance located in **Section 5.5** and standard therapy for acute drug reactions as per institutional standard of care and reported to the sponsor.

5.2 Dosing Criteria

The following dosing criteria must be met prior to the first dose each week after Week 1:

- ANC $\geq$1,000/mcL
- Platelets $\geq$90,000/mcL
- Hemoglobin $\geq$ 8 g/dL
- Total bilirubin $\leq$1.5 x ULN (patients with diagnosed Gilbert's Syndrome, direct bilirubin should be within normal institutional limits)
- AST(SGOT)/ALT(SGPT) $\leq$2.5 X ULN
- Creatinine $\leq$ 1.5 X institutional upper limit of normal or creatinine clearance $\geq$ 50 mL/min/1.73 m^2

5.3 Contraception, Use in Pregnancy, Use in Nursing

5.3.1 Contraception

Helixor® M may have adverse effects on a fetus *in utero*. Furthermore, it is not known if Helixor® M has transient adverse effects on the composition of sperm. Non-pregnant, non-breast-feeding women may be enrolled if they are considered highly unlikely to conceive. Highly unlikely to conceive is defined as 1) surgically sterilized, or 2) postmenopausal (a woman who is ≥45 years of age and has not had menses for greater than 2 years will be considered postmenopausal), or 3) amenorrheaic for <2 years without a hysterectomy and oophorectomy, or 4) not heterosexually active for the duration of the study, or 5) heterosexually active and willing to use a method of birth control (which is also required for the female partners of male patients). Birth control methods can be either a barrier method or a hormonal method to prevent pregnancy, used throughout the study starting with Visit 1 through 28 days after the last dose of study medication. Male patients enrolled in this study must also agree to use an adequate method of contraception starting with Visit 1 through 28 days after the last dose of study drug.

The following are considered adequate barrier methods of contraception: diaphragm, condom (by the partner), copper intrauterine device, sponge, or spermicide. Appropriate hormonal contraceptives will include any registered and marketed contraceptive agent that contains an estrogen and/or a progestational agent (including oral, subcutaneous, intrauterine, or intramuscular agents).

Patients should be informed that taking the study medication may involve unknown risks to the fetus (unborn baby) if pregnancy were to occur during the study. In order to participate in the study they must adhere to the contraception requirement (described above) for the duration of the study. If there is any question that a patient will not reliably comply with the requirements for contraception, that patient should not be entered into the study.

5.3.2 Use in Pregnancy

Helixor® M may have adverse effects on a fetus; therefore, women with a positive pregnancy test at screening will not be eligible for enrollment. If a patient inadvertently

becomes pregnant while on treatment with Helixor® M, the patient will immediately be removed from the study. The study team will contact the patient at least monthly and document the patient's status until the pregnancy has been completed or terminated. The outcome of the pregnancy will be reported to the Sponsor without delay. The outcome must be reported to the Sponsor within 24 hours if the outcome is a serious adverse experience (e.g., death, abortion, congenital anomaly, or other disabling or life-threatening complication to the mother or newborn). If a male patient's partner becomes pregnant on study the pregnancy must be reported to the Sponsor. The study Investigator will make every effort to obtain permission to follow the outcome of the pregnancy and report the condition of the fetus or newborn to the Sponsor.

5.3.3 Use in Nursing Women

It is unknown whether Helixor® M is excreted in human milk. Since many drugs are excreted in human milk, and because of the potential for serious adverse reactions in the nursing infant, patients who are breast-feeding are not eligible for enrollment.

5.4 Definition of Dose-Limiting Toxicity (DLT) and Maximum Tolerated Dose (MTD)

Dose limiting toxicities (DLTs) are defined as the following possibly, probably, or definitely related study drug toxicities:

- ≥ grade 3 systemic flu-like symptoms,
- Grade 3 or 4 febrile neutropenia
- Grade 3 or 4 thrombocytopenia with bleeding
- ≥ grade 3 non-hematologic and grade 4 hematologic toxicities, or
- Death
- Exceptions include
  - Asymptomatic laboratory abnormalities
  - Grade 3 fatigue
  - Grade 3 dermatologic AEs that are considered mild in severity but only considered grade 3 because of >30% body surface involvement (rash, pruritus, etc.)
  - Fever, chills rigors, hypertension, hypotension, syncope, or hypoxia that resolve within 24 hours of intervention
  - Diarrhea, nausea or vomiting that resolves to < grade 3 within 24 hours of intervention
  - Grade 3-4 hyperglycemia or grade 3 hypophysitis where symptoms are controlled on hormone replacement therapy

Patients will be observed for DLTs for 28 days after the first dose of Helixor® M. Patients who experience a DLT will be discontinued from study treatment. Any patient who receives < 75% of the scheduled doses during the DLT period but does not experience a DLT will be replaced.

This phase I study will evaluate up to 8 dose levels of Helixor® M as a single agent: 150 mg, 300 mg, 600 mg, 900 mg, 1200 mg, 1500 mg, 1800 mg, and 2000 mg. Dose escalation

will proceed using an accelerated titration design.

Patients will be assigned to a dose level in the order of study entry. The first cohort of 1 subject will receive mistletoe at the 150 mg dose level. One subject will be enrolled in each cohort until a subject experiences a drug-related toxicity of grader 2 or higher, whereupon an additional 2 patients will be enrolled at that dose level. Thereafter, the traditional 3+3 design will be instituted as described below, with 3 or 6 patients treated at this dose level and at all subsequent dose levels depending upon the incidence of DLTs.

**Number of Patients with DLT at a Given Dose Level**	**Escalation Decision Rule of the 3+3 design**
0 out of 3	Enter 3 patients at the next dose level.
≥2	Dose escalation will be stopped. This dose level will be declared the maximally administered dose (highest dose administered). Three (3) additional patients will be entered at the next lowest dose level if only 3 patients were treated previously at that dose.
1 out of 3	Enter at least 3 more patients at this dose level.<br>• If 0 of these 3 patients experience DLT, proceed to the next dose level.<br>• If 1 or more of this group suffer DLT, then dose escalation is stopped, and this dose is declared the maximally administered dose. Three (3) additional patients will be entered at the next lowest dose level if only 3 patients were treated previously at that dose.
≤1 out of 6 at highest dose level below the maximally administered dose	This is generally the recommended phase 2 dose. At least 6 patients must be entered at the recommended phase 2 dose.

The MTD is defined as the dose level immediately below the dose level at which ≥2 patients in a cohort (dose level) of 2 to 6 patients experienced a treatment-related DLT.

If MTDs are not determined at the dose of 2000 mg, this will be the dose taken to the expansion phase and no further escalation of dose is planned.

5.5 General Concomitant Medication and Supportive Care Guidelines

In general, concomitant medications and therapies deemed necessary for the supportive care and safety of the subject are allowed, provided their use is documented in the subject records and on the appropriate case report form. If toxicity occurs, the appropriate treatment will be used to ameliorate signs and symptoms [including antiemetics for nausea and vomiting, anti-diarrheals for diarrhea and anti-pyretics (only if ≥ 39°C or lasting more

than 3 days) and anti-histamines for drug fever]. All supportive measures for optimal medical care will be given during the period of study.

There is a possible risk of anaphylaxis and Helixor® M should be administered in a setting where emergency treatment is available. Administration should be immediately discontinued and the appropriate therapy instituted if there is a serious allergic reaction. The patient must be observed in the clinic for at least 30 minutes after the lead in dose and for the first dose of the assigned dose level. Acute reactions (allergic or allergic-like reactions such as generalized itching, hives, rash, swelling of the face or throat, chills, difficulty breathing, shock or spasm of the airways) will be managed using standard therapy for acute drug reactions as per institutional standard of care and reported to the sponsor. If a grade 1 or 2 hypersensitivity reaction is suspected, this must be discussed with the IND sponsor and principal investigator before the patient is given another dose of Helixor® M. Patients developing grade 3 or 4 hypersensitivity will not receive another dose of Helixor® M.

Guidance on Infusion/Hypersensitivity Reactions:

Treatment recommendations are provided below and may be modified based on local treatment standards and guidelines as appropriate:

For grade 1 symptoms (mild reaction; infusion interruption not indicated; intervention not indicated):

Remain at bedside and monitor subject until recovery from symptoms. The following prophylactic premedications are recommended for future infusions: diphenhydramine 50 mg (or equivalent) at least 30 minutes before additional Helixor® M administrations.

For grade 2 symptoms (moderate reaction requires therapy or infusion interruption but responds promptly to symptomatic treatment [e.g., antihistamines, non-steroidal anti-inflammatory drugs, narcotics, corticosteroids, bronchodilators, IV fluids]; prophylactic medications indicated for $\leq$ 24 hours):

Stop the Helixor® M infusion, begin an IV infusion of normal saline, and treat the subject with diphenhydramine 50 mg IV (or equivalent) and/or acetaminophen 325 to 1000 mg; remain at bedside and monitor subject until resolution of symptoms. If the infusion is interrupted, restart the infusion at 50% of the original infusion rate when symptoms resolve; if no further complications ensue after 30 minutes, the rate may be increased to 100% of the original infusion rate. Monitor subject closely. If symptoms recur then no further Helixor® M will be administered at that visit. Administer diphenhydramine 50 mg IV, and remain at bedside and monitor the subject until resolution of symptoms. The amount of study drug infused must be recorded on the case report form (CRF). The following prophylactic premedications are recommended for future infusions: diphenhydramine 50 mg (or equivalent) and/or acetaminophen 325 to 1000 mg should be administered at least 30 minutes before additional Helixor® M administrations.

For grade 3 or grade 4 symptoms (severe reaction, grade 3: prolonged [i.e., not rapidly responsive to symptomatic medication and/or brief interruption of infusion]; recurrence of

symptoms following initial improvement; hospitalization indicated for other clinical sequelae [e.g., renal impairment, pulmonary infiltrates]; grade 4: (life threatening; pressor or ventilator support indicated):

Immediately discontinue infusion of Helixor® M. Begin an IV infusion of normal saline, and treat the subject as follows. Recommend bronchodilators, epinephrine 0.2 to 1 mg of a 1:1,000 solution for subcutaneous administration or 0.1 to 0.25 mg of a 1:10,000 solution injected slowly for IV administration, and/or diphenhydramine 50 mg IV with methylprednisolone 100 mg IV (or equivalent), as needed. Subject should be monitored until the investigator is comfortable that the symptoms will not recur. Helixor® M will be permanently discontinued. Investigators should follow their institutional guidelines for the treatment of anaphylaxis. Remain at bedside and monitor subject until recovery from symptoms. In the case of late-occurring hypersensitivity symptoms (e.g., appearance of a localized or generalized pruritis within 1 week after treatment), symptomatic treatment may be given (e.g., oral antihistamine, or corticosteroids).

Helixor® M induced fever should not be treated with antipyrexia agents including but not limited to (Acetaminophen or NSAIDS, etc) if less than 38.5^{o}C. Prolonged fever beyond 3 days should be investigated for an alternative reason for fever.

Activation of pre-existing inflammatory irritation of superficial veins at the injection site may occur. In such cases, stop treatment temporarily until the inflammatory reaction has subsided.

The use of immunosuppressive systemic corticosteroids, immunosuppressive therapy, and growth factors including, but not limited to, granulocyte-colony stimulating factor (G-CSF), granulocyte macrophage-colony stimulating factor (GM-CSF), erythropoietin, etc. are prohibited while patients are on study. Patients are permitted to use topical, ocular, intra-articular, intranasal, and inhalational corticosteroids (with minimal systemic absorption). A brief course of corticosteroids for prophylaxis (e.g., contrast dye allergy) or for treatment of non-autoimmune conditions (e.g., delayed-type hypersensitivity reaction caused by contact allergen) is permitted. NSAIDs are to be avoided if possible. However, systemic steroid therapy is allowed for subjects on replacement therapy as long as prednisone ≤ 10 mg or its steroid equivalent.

5.6 Duration of Therapy

In the absence of treatment delays due to adverse event(s), treatment may continue until one of the following criteria applies:

- The patient or legal representative (such as a parent or legal guardian) withdraws consent for treatment
- Disease progression: Patients may be allowed to continue treatment if the Principal Investigator feels that the subject is receiving benefit based on the following criteria:
    - Investigator-assessed clinical benefit, and

- Subject is tolerating study drug.

The assessment of clinical benefit should take into account whether the subject is clinically deteriorating and unlikely to receive further benefit from continued treatment. The following criteria need to be taken into consideration:

- Absence of clinical symptoms and signs indicating disease progression.
- No decline in ECOG performance status.
- Absence of rapid progression of disease or of progressive tumor at critical anatomical sites requiring urgent alternative medical intervention.

All decisions to continue treatment beyond PD must be discussed with the Principal Investigator.

- Intercurrent illness that prevents further administration of treatment,
- DLT listed in **Section 5.4**,
- Grade 3 or 4 Helixor® M hypersensitivity reaction,
- Treatment delays lasting longer than 2 weeks due to a drug-related toxicity that does not resolve to a $\geq$ grade 1 or baseline despite supportive treatment per standard clinical practice,
- If in the opinion of the Investigator, a change or temporal or permanent discontinuation of therapy would be in the best interest of the patient,
- Noncompliance with trial treatment or procedure requirements,
- Patient is lost to follow-up, or
- Patient becomes pregnant.

5.7 Duration of Follow Up

Patients will be followed for 28 days after completion or removal from study or until death, whichever occurs first. Patients who are discontinued from the study treatment due to a drug-related AE will be monitored for safety until the resolution of the AE to $\leq$ grade 1 or stabilization or until initiation of a new therapy for their cancer, whichever occurs first.

Patients who have completed or discontinue from treatment should be contacted every six months to monitor overall survival. Information of other cancer therapies after discontinuation from the study treatment will be collected.

5.8 Criteria for Removal from Study

Patients will be removed from study when any of the criteria listed in **Section 5.6** applies. The reason for study removal and the date the patient was removed must be documented in the Case Report Form.

6. DOSING DELAYS AND SCHEDULING/DOSE MODIFICATIONS

6.1 Dosing Delays and Scheduling/Missed Doses

Helixor® M will be held for drug-related fever ≥ 39°C, chills, transient dizziness, or if the dosing criteria per **Section 5.2** are not met. If a delayed dose cannot be made up during the same week, it will be skipped. If toxicity does not resolve to ≤ grade 1 or baseline within 2 weeks, trial treatment should be discontinued after consultation with the IND sponsor and principal investigator.

Patient-initiated, pre-planned missed doses (for example, due to vacation plans) are not permitted during the first 8 weeks of treatment. Missed doses due to clinic scheduling and holiday closures are permitted. Patients are allowed to move 1 treatment per week by 1 day (for example: MWF →TWF). Helixor® M infusions may not be given less than 24 hours apart. In special cases, after a documented discussion with the PI, Helixor® M infusions may be given on three consecutive days (for example: MTW) as long as they are given at least 24 hours apart. Up to 3 missed doses of Helixor® M for every 12 doses will be permitted after week 8. Allowance of Helixor® M breaks of greater than 1 week will be decided on a case-by-case basis by the Principal Investigator.

6.2 Dose Modifications

When the delay is due to drug-related toxicity, the patient may resume Helixor® M with a dose reduction to the next lower dose level. Only two dose reductions due to the same toxicity will be permitted. In the event of a third occurrence of the same toxicity which would require a dose interruption, study therapy will be discontinued permanently. No dose reductions are permitted for patients enrolled in Dose Level 1.

7. ADVERSE EVENTS: LIST AND REPORTING REQUIREMENTS

This study will use the descriptions and grading scales found in the revised National Cancer Institute Common Terminology Criteria for Adverse Events (CTCAE) Version 4.03 for adverse event reporting that can be found at http://ctep.cancer.gov/protocolDevelopment/electronic_applications/ctc.htm.

The Investigator or designee must completely and promptly record each adverse event using the CRF, regardless of relationship to study drug as determined by the Investigator. The Investigator should attempt, if possible, to establish a diagnosis based on the patient's signs and symptoms. When a diagnosis for the reported signs or symptoms is known, the Investigator should report the diagnosis, not the symptoms, as the adverse event.

Information about all adverse events, whether volunteered by the subject, discovered by investigator questioning, or detected through physical examination, laboratory test or other means, will be collected, recorded, and followed as appropriate.

All adverse events experienced by patients will be collected and reported from the first dose of the investigational agent, throughout the study, and will only be followed for 28 days after the maximal planned dose is administered unless there are ongoing problems thought to be related to the investigational agent.

Patients who have an ongoing adverse event related to the study procedures and/or medication(s) may continue to be periodically contacted by a member of the study staff until the event is resolved or determined to be irreversible by the investigator.

Laboratory abnormalities: Laboratory abnormalities present at the screening visit will be recorded as pre-treatment signs and symptoms. After study treatment administration, all grade 3 and 4 clinical laboratory results that represent an increase in severity from baseline will be reported as adverse events. A grade 1 or 2 clinical laboratory abnormality should be reported as an adverse event only if it is considered clinically significant by the investigator.

7.1 Definitions

7.1.1 Adverse Event (AE)

Adverse event is defined as any undesirable sign, symptom or medical condition occurring after starting the study drug (or therapy) even if the event is not considered to be related to the study. An undesirable medical condition can be symptoms (e.g., nausea, chest pain), signs (e.g., tachycardia, enlarged liver) or the abnormal results of an investigation (e.g., laboratory findings, electrocardiogram). Medical conditions/diseases present before starting the study treatment are only considered adverse events if they worsen after starting the study treatment (any procedures specified in the protocol). Adverse events occurring before starting the study treatment but after signing the informed consent form will not be recorded. Abnormal laboratory values or test results constitute adverse events only if they induce clinical signs or symptoms or require therapy.

7.1.2 Serious Adverse Event (SAE)

A serious adverse event is an undesirable sign, symptom or medical condition which:

- Results in death
- Is life threatening (defined as an event in which the subject was at risk of death at the time of the event; it does not refer to an event which hypothetically might have caused death if it were more severe)
- Requires inpatient hospitalization or causes prolongation of existing hospitalization (see note below for exceptions) for ≥ 24 hours
- Results in persistent or significant disability/incapacity
- Is a congenital anomaly/birth defect (note: reports of congenital anomalies/birth defects must also be reported on the Pregnancy Supplemental Form)
- Is an important medical event (defined as a medical event(s) that may not be immediately life-threatening or result in death or hospitalization but, based upon appropriate medical and scientific judgment, may jeopardize the subject or may require intervention [e.g., medical, surgical] to prevent one of the other serious

outcomes listed in the definition above.). Examples of such events include, but are not limited to, intensive treatment in an emergency room or at home for allergic bronchospasm; blood dyscrasias or convulsions that do not result in hospitalization.)

Events **not** considered to be serious adverse events are hospitalizations for the:
- Admissions as per protocol for a planned medical/surgical procedure or to facilitate a procedure
- Routine health assessment requiring admission for baseline/trending of health status (e.g., routine colonoscopy)
- Medical/surgical admission for purpose other than remedying ill health state and was planned prior to entry into the study. Appropriate documentation is required in these cases
- Admission encountered for another life circumstance that carries no bearing on health status and requires no medical/surgical intervention (e.g., lack of housing, economic inadequacy, care-giver respite, family circumstances, administrative).

7.2 Relationship

Definite – The AE *is clearly related* to the study treatment.
Probable – The AE *is likely related* to the study treatment.
Possible – The AE *may be related* to the study treatment.
Unlikely – The AE *is doubtfully related* to the study treatment.
Unrelated – The AE *is clearly NOT related* to the study treatment.

7.3 Expectedness

Unexpected adverse event: An adverse event, which varies in nature, intensity or frequency from information on the investigational drug/agent provided in the Investigator's Brochure, package insert or safety reports. Any adverse event that is not included in the informed consent is considered "unexpected".

Expected (known) adverse event: An adverse event, which has been reported in the Investigator's Brochure. An adverse event is considered "expected", only if it is included in the informed consent document as a risk.

7.4 Handling of Expedited Safety Reports

In accordance with local regulations, the IND Sponsor (Dr. Paller) will notify investigators of all SAEs that are unexpected (ie, not previously described in the package insert or Investigator Brochure), and definitely, probably, or possibly related to Helixor® M. This notification will be in the form of an expedited safety report (ESR) that is to be faxed to the investigators and the study coordinators within 24 hours of notification of the event. Upon receiving such notices, the investigator must review and retain the notice with the Investigator's Brochure and where required by local regulations, the investigator will submit the ESR to the appropriate IRB. The investigator and IRB will determine if the informed consent requires revision. The investigator should also comply with the IRB

procedures for reporting any other safety information. Where required, submission of ESRs by the investigator to Health Authorities should be handled according to local regulations.

7.5 Reporting

7.5.1 General

All adverse events (both expected and unexpected) will be captured on the appropriate study-specific case report forms (CRFs). In addition, all serious adverse events, regardless of causality to study drug, will be reported promptly to the IND Sponsor Dr. Paller (410-955-8239, email: cpaller1@jhmi.edu) within 24 hours of recognition of the adverse event using the form found in **Appendix B**. If this falls on a weekend or holiday, an email notification is acceptable but must be followed by an SAE reporting form on the next business day.

7.5.2 Institutional Review Board (IRB)

All serious adverse events will be reported to the Institutional Review Board (IRB) per institutional guidelines. Follow-up information will be submitted to the IRB per institutional guidelines as soon as relevant information is available.

7.5.3 Food and Drug Administration (FDA)

7.5.3.1 Expedited IND Safety Reports:

7 Calendar-Day Telephone or Fax Report:
The IND Sponsor is required to notify the FDA of any fatal or life-threatening adverse event that is unexpected and assessed by the investigator to be possibly related to the investigational agent. Such reports are to be telephoned or faxed (301-796-9845) to the FDA within 7 calendar days of first learning of the event. Follow-up information will be submitted to the FDA as soon as relevant information is available.

15 Calendar-Day Written Report:
The IND Sponsor is required to notify the FDA of any serious adverse event that is unexpected and possibly related to the investigational agent in a written IND Safety Report.

Written IND Safety Reports should include an Analysis of Similar Events in accordance with regulation 21 CFR § 312.32. All safety reports previously filed with the IND concerning similar events should be analyzed. The new report should contain comments on the significance of the new event in light of the previous, similar reports.

Written IND safety reports with Analysis of Similar Events are to be submitted to the FDA within 15 calendar days of first learning of the event. Follow-up information will be submitted to the FDA as soon as relevant information is available.

7.5.3.2 IND Annual Reports

In accordance with the regulation 21 CFR § 312.33, the IND Sponsor shall within 60 days of the anniversary date that the IND went into effect submit a brief report of the adverse events and progress of the investigation. Please refer to Code of Federal Regulations, 21 CFR § 312.33 for a list of the elements required for the annual report. All IND annual reports will be submitted to the FDA by the IND Sponsor.

7.6 Possible Adverse Events

Adverse drug reactions to Helixor IV infusion include urticaria, generalized or localized itching, dyspnea/bronchospasm, quincke's edema, activation of former SC injection site reactions, erythema with or without swelling, fever, exanthema, anaphylaxis, chills, flu-like symptoms, abdominal pain, joint swelling, paresthesia, deterioration of pre-existing bone pain. Additional reported side effects include: fatigue, headache, and infusion site inflammation.

8. PHARMACEUTICAL INFORMATION

A list of the adverse events and potential risks associated with Helixor® M can be found in **Section 7.6**.

Detailed instructions regarding the storage, preparation, and administration of Helixor® M can be found in the pharmacy manual.

8.1 Mode of Action

Several biologically active substances are found in mistletoe extracts: lectins, viscotoxins, peptides, amino acids, flavonoides, oligo and polysaccharides, and other substances in low concentrations[2]. Mistletoe extract has two mechanisms of action, tumor cytotoxicity and immunomodulation. The cytotoxic effects of mistletoe extract are a result of protein synthesis interference[3,4], cell cycle inhibition[5], and inducing apoptosis[5-8]. The immunomodulatory properties of mistletoe extract work through increasing white blood cell numbers, as well as stimulating the production of immune enhancing cytokines (IL-1, IL-6, TNF-α)[9,10]. Lastly, mistletoe extract has been shown to have anti-angiogenic properties[11]. In Europe, Helixor® M is used as an anthroposophic medicine in the treatment of malignant and non-malignant tumors, malignant disorders with accompanying impairment of the hematopoietic organs, stimulation of bone marrow activity, reducing the risk of tumor recurrence, and diagnosed pre-cancerous disorders.

8.2 Product description

1 ml injection solution Helixor® M 50 mg contain: 1,006 mg aqueous extract (1:20) from 50 mg fresh herbal substance of apple tree mistletoe. Helixor® M 100 mg has the same strength but is filled in 2-ml ampoules. Other constituents include: Sodium chloride for isotonization, sodium hydroxide for pH adjustment. Water for injection is added as vehicle for the medicinal product formulation.

Sterile injection solution filled into burn-up ampoules with single point cut (DIN ISO 9187) made of colorless glass type I. Supplied as Original Packs (OPs) each with 8 ampoules Helixor® M or as Great Packs (GPs) each with 50 ampoules Helixor® M.

8.3 Storage and Labeling

The product should be stored in the refrigerator (5 °C ± 3 °C) in the original package to protect the contents from light. The ampoules should not be used if they have been previously opened or exposed to ultraviolet light.

8.4 Stability

Helixor® M has a shelf life of 2 years; the expiration date is printed on each ampoule and box. The administration of Helixor® M must be completed within 24 hours of preparation. If not used immediately, the infusion solution may be stored under refrigeration conditions (2°-8°C, 36°-46°F) for up to 20 hours, and a maximum of 4 hours of the total 24 hours can be at room temperature (20°-25°C, 68°-77°F). The maximum 4-hour period under room temperature includes the product administration period. The IV bag should be allowed to equilibrate to room temperature prior to subsequent use.

8.5 Preparation and Administration

Helixor® M should be diluted with 0.9% Sodium Chloride Injection, USP using the following Reconstitution schema:

Dose Group (mg)	Investigational Product	0.9% NaCl
50	1 ampule (1ml)	249ml
150	1.5 ampules (3ml)	247ml
300	3 ampules (6ml)	244ml
600	6 ampules (12ml)	238ml
900	9 ampules (18ml)	232ml
1200	12 ampules (24ml)	226ml
1500	15 ampules (30ml)	220ml
1800	18 ampules (36ml)	214ml
2000	20 ampules (40ml)	210ml

The administration of Helixor® M at room temperature (20°-25°C, 68°-77°F) must be completed within 4 hours. Care must be taken to assure sterility of the prepared solution as the product does not contain any anti-microbial preservative or bacteriostatic agent. In this study, Helixor® M will be administered intravenously three times per week. To avoid pseudo-allergic reaction the optimum drip speed should not be exceed 26 drops per minute, corresponding to the infusion time of approximately 3 hours. Infusion times are approximate (+/- 15min) and may need to be adjusted based on patient tolerability. At the end of the infusion, flush the line with a sufficient quantity of normal saline.

8.6 Patient Care Implications

There is a possible risk of anaphylaxis and Helixor® M should be administered in a setting where emergency treatment is available. Administration should be immediately discontinued and the appropriate therapy instituted if there is a serious allergic reaction. The patient must be observed in the clinic for at least 30 minutes after the lead in dose and the first dose of the assigned dose level. Acute reactions (allergic or allergic-like reactions such as generalized itching, hives, rash, swelling of the face or throat, chills, difficulty breathing, shock or spasm of the airways) will be managed using standard therapy for acute drug reactions as per institutional standard of care and reported to the sponsor. If a grade 1 or 2 hypersensitivity reaction is suspected, this must be discussed with the IND sponsor and principal investigator before the patient is given another dose of Helixor® M. Patients developing grade 3 or 4 hypersensitivity will not receive another dose of Helixor® M.

Interactions of mistletoe products with other drugs are not known. In two different studies, neither induction nor inhibition of cytochrome P450 isoenzymes could be detected for Helixor® M.

8.7 Returns and Reconciliation

The investigator is responsible for keeping accurate records of the clinical supplies received from Helixor or designee, the amount dispensed to, and returned by the subjects and the amount remaining at the conclusion of the trial.

Upon completion or termination of the study, all unused and/or partially used investigational product will be destroyed at the site per institutional policy. It is the Investigator's responsibility to arrange for disposal of all empty containers, provided that procedures for proper disposal have been established according to applicable federal, state, local and institutional guidelines and procedures, and provided that appropriate records of disposal are kept.

9. CORRELATIVE/SPECIAL STUDIES

9.1 Peripheral Blood Mononuclear Cells (PBMC) Studies

Baseline and post-treatment PBMC will be collected to monitor peripheral immunologic changes in response to therapy. The peripheral blood will be obtained from each research participant at periodic protocol-specified intervals for *in vitro* assays, such as ELISPOT assays against CEF (Influenza, CMV, and EBV) peptide pools. Up to 32 ml of blood will be obtained from all eligible patients at the designated time points in 8 ml CPT tubes. PBMC will be processed by standard laboratory procedures, transferred to cryogenic vials, and stored in a liquid nitrogen freezer until further analysis. Please see the Laboratory Manual for further details.

9.2 Serum Studies

Baseline and post-treatment sera will be collected to identify potential therapeutic targets, biomarkers, and predictors of response. Serology analysis for cytokine production by PBMC will be evaluated using the human serum cytokine multiplex approach that can test for up to 27 different cytokines (IL-1b, IL-1ra, IL-2, IL-4, IL-5, IL-6, IL-7, IL-8, IL-9, IL-

10, IL-12(p70), IL-13, IL-15, IL-17, eotaxin, basic FGF, G-CSF, GMCSF, IFNγ, IP-10, MCP-1, MIP1a, MIP1b, PDGF-BB, VEGF, TNFα, and TGFβ). Whole blood (up to 10 ml) will be collected in a 10 milliliter Serum Separator Tube (SST tube) at the designated time points. Aliquots of 1 ml of serum will be transferred to cryogenic vials and stored at -80°C or below. Please see the Laboratory Manual for further details.

9.3 Plasma Studies

Baseline and post-treatment plasma will be collected to assess tumor burden dynamics using exploratory circulating biomarkers, as well as to evaluate germline mutations in circulating DNA that may correlate with clinical benefit. Whole blood (up to 20 ml) will be collected at the designated time points in a 10 ml plasma preparation tubes with EDTA (PPT, BD Vacutainer, Franklin Lakes, NJ) and gently swirled to mix blood with EDTA. Within three hours of collection, the sample will be processed using standard procedures for plasma separation. Plasma will be divided into 1 ml aliquots and stored at -80°C. Pellets from this separation procedure will also be stored at -80°C. Please see the Laboratory Manual for further details.

10. STUDY CALENDAR

	Pre-Study	Week 1[11]			Weeks 2-3			Week 4			Weeks 5-7			Week 8			Weeks 9-11			Week 12			Weeks 13-15			Week 16[12]			Off study[13]
		M	W	F	M	W	F	M	W	F	M	W	F	M	W	F	M	W	F	M	W	F	M	W	F	M	W	F	
Visit Windows (days)[1]	-28 to -1	+2	-	-	+2	-	-	+2	-	-	+2	-	-	+2	-	-	+2	-	-	+2	-	-	+2	-	-	+2	-	-	+/- 7
**Helixor® M**[2]		X----------																										----------X	
Informed consent	X																												
Eligibility criteria	X																												
Demographics	X																												
Medical and Cancer History	X																												
Con meds	X	X ----------																										---------- X	X
Physical exam[3]	X	X						X						X						X						X			X
Vital signs[4]	X	X	X	X	X	X	X	X	X	X	X	X	X	X	X	X	X	X	X	X	X	X	X	X	X	X	X	X	X
Height[4]	X																												
Weight	X	X						X						X						X						X			X
Performance status	X	X						X						X						X						X			X
FACT-G Questionnaire		X						X						X						X						X			X
Hematology, Chemistry profile [5,8]	X	X			X			X			X			X			X			X			X			X			X
Urinalysis & Microscopic Exam[6,8]	X																												
Pregnancy test[7,8]	X	X						X						X						X						X			
Relevant Tumor Marker[5,8]	X	X						X						X						X						X			X
Adverse event evaluation		X ----------																										---------- X	X
Radiologic evaluation[9]	X													X												X			X

	Pre-Study	Week 1[11]			Weeks 2-3			Week 4			Weeks 5-7			Week 8			Weeks 9-11			Week 12			Weeks 13-15			Week 16[12]			Off study[13]
		M	W	F	M	W	F	M	W	F	M	W	F	M	W	F	M	W	F	M	W	F	M	W	F	M	W	F	
Tumor measurements[9]	X													X												X			X
PBMC (up to 32ml)		X						X						X						X						X			X
Serum (up to 10ml)		X						X						X						X						X			X
Whole Blood for Plasma (up to 20ml)		X						X						X						X						X			X
Archival Tissue[10]	X (optional with consent)																												

1: Longer delays to be approved by the IND sponsor. Patient-initiated dosing delays are not permitted during the first 8 weeks of treatment. Dosing delays due to clinic scheduling and holiday closures are permitted. Patients are allowed to move 1 treatment per week by 1 day (for example: MWF →TWF). Helixor® M infusions may not be given less than 24 hours apart.

2: Please see **Section 5.1** for dosing schedule according to the assigned dose level

3: Complete physical exam will be completed at baseline; focused physical examinations will be conducted thereafter. If study drug is held due to an AE, a physical exam is required before patient is allowed to restart drug.

4: Temperature, respiration rate, blood pressure, and pulse should be taken at the baseline and prior to each administration of Helixor® M. The patient must be observed in the clinic for at least 30 minutes after the lead in dose and the first dose of the assigned dose level. Height will be taken at or prior to screening only.

5: Clinical hematology: CBC with differential and platelet count; serum chemistry: sodium, potassium, chloride, bicarbonate, glucose, BUN, creatinine, ALT, AST, alkaline phosphatase, total bilirubin, total protein, albumin, and calcium. Clinically relevant tumor markers may be drawn by the clinician. For example, prostate cancer patients get monthly PSA values.

6: Bilirubin, blood, glucose, ketones, leukocytes, nitrite, pH, protein, and specific gravity

7: Pregnancy tests will be administered to WOCBP: Serum pregnancy test at baseline and urine pregnancy test thereafter.

8: Labs may be collected within a window of up to 3 days prior to dosing.

9: Radiologic evaluations (CT scan of the chest, abdomen, and pelvis or MRI if patient has contrast allergy) and tumor measurements (RECIST 1.1 and irRC) will be performed at baseline, week 8 (+/-7 days), and every 8 weeks (+/-7 days) thereafter. Off study scans only need to be completed if the off study visit occurs before the Week 8 scan; scans do not need to be repeated if one has been done within the past 6 weeks. Bone scans will also be performed if applicable.

10: Attempts to obtain surgical or biopsy archival tumor samples will be made for every subject who consents to the optional sample until the sample is obtained or documentation that the sample cannot be obtained. Detailed instructions for tissue collection, processing and shipment are provided in the

Laboratory Manual.

11: For Cycle 1 Day 1 (50mg dose level) study procedures do not need to be repeated if they were conducted within 3 days of the pre-study evaluations.

12: Beyond Week 16, repeat the study assessments and evaluations detailed in Weeks 9-16 until one of the criteria from **Section 5.6** applies.

13: 28 days after their last dose of study drug or within 7 days prior to initiation of a new anti-cancer treatment, whichever comes first. Patients who have completed or discontinue from treatment should be contacted every six months (+/- 28 days) to monitor overall survival. Information regarding anti-cancer therapies after discontinuation from the study treatment will also be collected.

11. MEASUREMENT OF EFFECT

Although response is not the primary endpoint of this trial, patients with measurable disease will be assessed by standard criteria. For the purposes of this study, patients should be evaluated at baseline, week 8, every 8 weeks thereafter, and at the off study visit (if the patient comes off study before week 8 or it has been more than 6 weeks since the last scan) with a CT with oral and IV contrast of the chest, abdomen and pelvis (or MRI if patient has contrast allergy). In addition a bone scan will be performed for prostate cancer patients at baseline and at study completion.

11.1 Antitumor Effect – Solid Tumors

Response and progression will be evaluated in this study using the new international criteria proposed by the revised Response Evaluation Criteria in Solid Tumors (RECIST) guideline (version 1.1) [**Appendix D**] and the immune related Response criteria (irRC) [**Appendix E**]. Clinical decisions regarding disease progression will be based on RECIST criteria. Changes in the largest diameter (unidimensional measurement) of the tumor lesions and the shortest diameter in the case of malignant lymph nodes are used in the RECIST criteria.

11.1.1 Definitions

Evaluable for toxicity. All patients will be evaluable for toxicity from the time of their first treatment with Mistletoe.

Evaluable for objective response. Only those patients who have measurable disease present at baseline and have received at least one dose of therapy will be considered evaluable for response. These patients will have their response classified according to the definitions stated below.

Evaluable Non-Target Disease Response. Patients who have lesions present at baseline that are evaluable but do not meet the definitions of measurable disease and have received at least one dose of therapy will be considered evaluable for non-target disease. The response assessment is based on the presence, absence, or unequivocal progression of the lesions.

11.1.2 Methods for Evaluation of Measurable Disease

All measurements should be taken and recorded in metric notation using a ruler or calipers. All baseline evaluations should be performed as closely as possible to the beginning of treatment and never more than 4 weeks before the beginning of the treatment.

The same method of assessment and the same technique should be used to characterize each identified and reported lesion at baseline and during follow-up. Imaging-based evaluation is preferred to evaluation by clinical examination unless the lesion(s) being followed cannot be imaged but are assessable by clinical exam.

Conventional CT and MRI This guideline has defined measurability of lesions on CT scan based on the assumption that CT slice thickness is 5 mm or less. If CT scans have slice thickness greater than 5 mm, the minimum size for a measurable lesion should be twice the slice thickness. MRI is also acceptable in certain situations (e.g. for body scans).

Use of MRI remains a complex issue. MRI has excellent contrast, spatial, and temporal resolution; however, there are many image acquisition variables involved in MRI, which greatly impact image quality, lesion conspicuity, and measurement. Furthermore, the availability of MRI is variable globally. As with CT, if an MRI is performed, the technical specifications of the scanning sequences used should be optimized for the evaluation of the type and site of disease. Furthermore, as with CT, the modality used at follow-up should be the same as was used at baseline and the lesions should be measured/assessed on the same pulse sequence. It is beyond the scope of the RECIST guidelines to prescribe specific MRI pulse sequence parameters for all scanners, body parts, and diseases. Ideally, the same type of scanner should be used and the image acquisition protocol should be followed as closely as possible to prior scans. Body scans should be performed with breath-hold scanning techniques, if possible.

11.1.3 Duration of Response

Duration of overall response: The duration of overall response is measured from the time measurement criteria are met for CR or PR (whichever is first recorded) until the first date that recurrent or progressive disease is objectively documented (taking as reference for progressive disease the smallest measurements recorded since the treatment started).

The duration of overall CR is measured from the time measurement criteria are first met for CR until the first date that progressive disease is objectively documented.

Duration of stable disease: Stable disease is measured from the start of the treatment until the criteria for progression are met, taking as reference the smallest measurements recorded since the treatment started, including the baseline measurements.

11.1.4 Progression-Free Survival (PFS)

PFS is defined as the duration of time from start of treatment to time of first documented progression of disease or death from any cause, whichever occurs first.

11.1.5 Time to Progression (TTP)

TTP is defined as the time from start of treatment to the time of progression. Deaths (all cause) are censored at time of death.

12. DATA REPORTING / REGULATORY REQUIREMENTS

Adverse event lists, guidelines, and instructions for AE reporting can be found in **Section 7.0** (Adverse Events: List and Reporting Requirements).

12.1 Data Management

All information will be collected on study-specific case report forms (CRFs) by study staff. These data will be reviewed for completeness and accuracy by the Principal Investigator at each site.

Protocol Chair
The Protocol Chair is responsible for performing the following tasks:
- Coordinating, developing, submitting, and obtaining approval for the protocol as well as its subsequent amendments.
- Assuring that all participating institutions are using the correct version of the protocol.
- Taking responsibility for the overall conduct of the study at all participating institutions and for monitoring the progress of the study.
- Reviewing and ensuring reporting of Serious Adverse Events (SAE)
- Reviewing data from all sites.

Coordinating Center
The Coordinating Center is responsible for performing the following tasks:
- Ensuring that IRB approval has been obtained at each participating site prior to the first patient registration at that site, and maintaining copies of IRB approvals from each site.
- Managing central patient registration.
- Collecting and compiling data from each site.
- Establishing procedures for documentation, reporting, and submitting of AE's and SAE's to the Protocol Chair, and all applicable parties.
- Facilitating audits by securing selected source documents and research records from participating sites for audit, or by auditing at participating sites.

Participating Sites
Participating sites are responsible for performing the following tasks:
- Following the protocol as written, and the guidelines of Good Clinical Practice (GCP).
- Submitting data to the Coordinating Center.
- Registering all patients with the Coordinating Center by submitting patient registration form, and signed informed consent promptly.
- Providing sufficient experienced clinical and administrative staff and adequate facilities and equipment to conduct a collaborative trial according to the protocol.
- Maintaining regulatory binders on site and providing copies of all required documents to the Coordinating Center.
- Collecting and submitting data according to the schedule specified by the protocol.

12.2 Safety Meetings

Scheduled meetings will take place bi-weekly and will include the protocol principal investigator, study coordinator(s), data manager(s), sub-investigators (as appropriate), collaborators (as appropriate), and biostatisticians (as appropriate) involved with the conduct of the protocol. During these meetings matters related to the following will be discussed: safety of protocol participants, validity and integrity of the data, enrollment rate relative to expectation, characteristics of participants, retention of participants, adherence to protocol (potential or real protocol violations), data completeness, and progress of data for objectives.

Teleconferences will be scheduled to include the Investigator and/or representatives from each site based on patient enrollment. During these meetings, the Investigators will discuss progress updates with a focus on safety of the protocol participants, enrollment status, and progress of data for objectives.

12.3 Monitoring

This is a DSMP Level II study under the SKCCC Data Safety Monitoring Plan (DSMP, 12/6/2012). Eligibility for all sites will be monitored by the Protocol chair. Data monitoring of this protocol will occur on a regular basis with the frequency dependent on the rate of subject accrual and the progress of the study. The protocol will be monitored internally by the Principal Investigator at each site. Additional data and safety monitoring oversight will also be performed by the SKCCC Safety Monitoring Committee (SMC - as defined in the DSMP). External monitoring will occur according to the following guidelines:

Johns Hopkins SKCCC: The protocol will be monitored externally by the SKCCC CRO in accordance with SKCCC guidelines. Trial monitoring and reporting will be done through the Compliance Monitoring Office at SKCCC.

Participating site(s): The protocol will be monitored by the internal CRO at each site. A report of the reviews will be submitted to the Johns Hopkins principal investigator and SKCCC CRO.

Authorized representatives of the Coordinating Center may visit the satellite sites to perform audits or inspections, including source data verification. The purpose of these audits or inspections is to systematically and independently examine all trial-related activities and documents to determine whether these activities were conducted and data were recorded, analyzed, and accurately reported according to the protocol, Good Clinical Practice (GCP), and any applicable regulatory requirements.

13. STATISTICAL CONSIDERATIONS

13.1 Study Design/Endpoints

This phase I study will evaluate up to 8 dose levels of Helixor® M as a single agent to determine the MTD of Helixor® M in patients with advanced solid tumors. The Helixor® M study dose levels are 150 mg, 300 mg, 600 mg, 900 mg, 1200 mg, 1500 mg, 1800 mg,

and 2000 mg. Patients will be assigned to a dose level in the order of study entry. If MTDs are not determined at the dose of 2000 mg, this will be the dose taken to the expansion phase and no further escalation of dose is planned.

An accelerated titration design (ATD) will be utilized to determine the MTD. The first cohort of 1 subject will receive Helixor® M at the 150mg dose level. One subject will be enrolled in each cohort until a subject experiences a grade 2 or higher drug related toxicity, whereupon an additional 2 patients will be enrolled at that dose level. Patients will be observed for DLTs through the first three doses at the highest dose per dose level (i.e.150 mg for DL1, 300 mg for DL2, 600 mg for DL3, etc.). Thereafter, the study will become the traditional 3 + 3 design with 3 or 6 patients treated at this dose level and at all subsequent dose levels depending upon the incidence of DLTs.

If no DLTs occur in a cohort of 3 patients, a new cohort of 3 patients will be treated at the next higher dose level. If 1 of 3 patients in a cohort experiences a DLT, that cohort will be expanded to 6 patients. If only 1 of the 6 patients experiences a DLT, then the next cohort of 3 patients will be treated at the next higher dose level. If 2 or more DLTs occur within a cohort, then that dose level will be above the maximum tolerated dose, and new patients will be enrolled at the previous lower (tolerated) dose level until that cohort has 6 patients. A total of 6 patients must be enrolled at a dose level in order to declare MTD. If ≤ 1 in 6 patients experience DLT at a dose level, dose escalation will stop and this dose level will be the recommended dose for the expansion phase.

The target DLT rate is < 33%. The MTD will be defined as the dose of Helixor® M in which < 2 of 6 patients experiences a dose limiting toxicity (DLT) with the next higher dose having at least 2 of 3 or 2 of up to 6 patients experiencing a DLT. A subject who is withdrawn from the study prior to completion of the DLT period for a reason other than a DLT and has not experienced a study drug-related adverse event of ≥ grade 2 may be replaced.

Once the MTD (or maximum administered dose if no MTD is determined) has been defined, an additional 10 patients will be enrolled and treated at that dose level in an expansion phase (for a total of 16 patients treated with the MTD) to further characterize safety and obtain preliminary estimates of efficacy.

Continuous evaluation of toxicity events in the expansion cohort will be performed throughout enrollment. If the rate of toxicities meeting DLT criteria appears to be higher than 30%, we will temporarily halt the study pending dose modification. Specifically, we will apply a Bayesian toxicity monitoring rule that suspends the enrollment if the posterior probability of risk being larger than 0.3 is 75% or higher.

We expect the SAE rate is about 27%. The monitoring rule uses beta(2, 5.5) as a prior distribution. This means that our prior guess at the proportion of toxicity is 27%, and there is 90% probability that this proportion is between 6% and 55%.

The decision rule for safety stopping is as follows:
Stop if:

# AE	2	3	4	5
**Out of**	2	3	6	9
		4	7	10
		5	8	

For example, if two out of the first 2 patients have adverse events, we will stop the accrual. If three or more out of the first 3-5 patients have adverse events, we will stop.

The operating characteristics of the stopping rule are shown below and are based on 5000 simulations:

True AE rate	% Simulated trials declaring unsafe	Average sample size (out of 10)
0.10	5.1	9.7
0.20	10.8	9.4
0.25	17.9	9.1
0.30	28.9	8.5
0.35	39.8	8.0
0.40	51.6	7.4
0.45	62.8	6.7

13.2 Sample Size/Accrual Rate

The phase I dose escalation study will require a minimum of 3 patients and a maximum 48 of patients. The expansion phase will evaluate 16 patients total including the 6 patients that are treated at MTD or maximum administered dose in dose escalation portion.

13.3 Analysis of Primary/Secondary Endpoints

The primary objective is to determine the safety, to characterize the toxicity profile of Helixor® M administered IV in patients with advanced solid tumors and to identify the maximum tolerated dose (MTD). Safety assessment measures of toxicity and adverse events will be tabulated by type and grade and will be objectively reviewed to determine the safety and tolerance of Helixor® M. The MTD will be defined as the dose of Helixor® M in which < 2 of 6 patients experiences a dose limiting toxicity (DLT) with the next higher dose having at least 2 of 3 or 2 of up to 6 patients experiencing a DLT.

In the expansion cohort, we will estimate the objective response rate (ORR) for the 16 patients. If we observe 3 responses (19%), the corresponding 90% confidence interval is [7%, 39%].

Secondary endpoints will be to measure tumor marker dynamics in patients receiving treatment. Tumor marker dynamics will be presented with summary statistics and boxplots.

Exploratory objectives will be to 1) to estimate PFS and TTP in patients treated with Helixor® M at MTD or maximum administered dose if no MTD is determined, and to measure tumor marker dynamics in patients receiving treatment, 2) collect peripheral blood mononuclear cells (PBMCs) and serum to explore potential therapeutic targets, biomarkers, and predictors of treatment response, 3) assess tumor burden dynamics using exploratory circulating biomarkers in serial collections of sera and plasma at baseline and throughout treatment, 4) collect pre and post-treatment whole blood to evaluate germline mutations in circulating DNA that may correlate with clinical benefit, and 5) determine changes in cytokine production by peripheral blood mononuclear cells (PBMC) as indicated by interleukin 2 [IL-2], interleukin 6 [IL-6], interleukin 8 [IL-8], interleukin 12 [IL-12], and interferon- gamma [IFN-γ]. PFS and TTP will be analyzed as time-to-event outcomes, for which we can estimate event-time distributions using the Kaplan-Meier method. PFS will be calculated as the time from start of treatment to time of progression per RECIST 1.1 and irRC or death, whichever occurs first. If the subject does not have a documented date of progression or death, PFS will be censored at the date of the last adequate assessment. Patients who start any subsequent anti-cancer therapy without a prior reported progression will be censored at the last evaluable tumor assessment prior to initiation of the subsequent anti-cancer therapy. TTP is the time from start of treatment to time of progression per RECIST 1.1 and irRC.

We will examine the correlation between potential therapeutic targets/biomarkers/predictors of treatment response and clinical endpoints. For time-to-event clinical endpoints, the potential predictors will be treated as covariates (time-varying or not) in Cox proportional-hazard models. For other clinical endpoints, we will use generalized estimating equations (GEE) to analyze the correlation between the potential predictors and clinical endpoints. Summary statistics and boxplots will be used to present the levels of potential predictors at each time point, assess tumor burden dynamics, compare pre and post-treatment germline mutations in circulating DNA, and examine the changes in cytokine production by PBMC.

13.4 Reporting and Exclusions

13.4.1 Evaluation of toxicity – All patients will be evaluable for toxicity from the time of their first treatment with Mistletoe.

13.4.2 Evaluation of response – All patients included in the study must be assessed for response to treatment, even if there are major protocol treatment deviations or if they are ineligible. Each patient will be assigned one of the following categories: 1) complete response, 2) partial response, 3) stable disease, 4) progressive disease, 5) early death from malignant disease, 6) early death from toxicity, 7) early death because of other cause, or 9) unknown (not assessable, insufficient data). [Note: By arbitrary convention, category 9 usually designates the "unknown" status of any type of data in a clinical database.]

All of the patients who met the eligibility criteria (with the possible exception of those who received no study medication) should be included in the main analysis of the response rate. Patients in response categories 4-9 should be considered to have a treatment failure (disease progression). Thus, an incorrect treatment

schedule or drug administration does not result in exclusion from the analysis of the response rate. Precise definitions for categories 4-9 will be protocol specific.

All conclusions should be based on all eligible patients. Subanalyses may then be performed on the basis of a subset of patients, excluding those for whom major protocol deviations have been identified (e.g., early death due to other reasons, early discontinuation of treatment, major protocol violations, etc.). However, these subanalyses may not serve as the basis for drawing conclusions concerning treatment efficacy, and the reasons for excluding patients from the analysis should be clearly reported. The 95% confidence intervals should also be provided.

REFERENCES

1. Mansky PJ. Mistletoe and cancer: controversies and perspectives. Seminars in oncology 2002;29:589-94.
2. Horneber MA, Bueschel G, Huber R, Linde K, Rostock M. Mistletoe therapy in oncology. The Cochrane database of systematic reviews 2008:CD003297.
3. Stirpe F, Sandvig K, Olsnes S, Pihl A. Action of viscumin, a toxic lectin from mistletoe, on cells in culture. The Journal of biological chemistry 1982;257:13271-7.
4. Sweeney EC, Palmer RA, Pfuller U. Crystallization of the ribosome inactivating protein ML1 from Viscum album (mistletoe) complexed with beta-D-galactose. Journal of molecular biology 1993;234:1279-81.
5. Harmsma M, Gromme M, Ummelen M, Dignef W, Tusenius KJ, Ramaekers FC. Differential effects of Viscum album extract IscadorQu on cell cycle progression and apoptosis in cancer cells. International journal of oncology 2004;25:1521-9.
6. Janssen O, Scheffler A, Kabelitz D. In vitro effects of mistletoe extracts and mistletoe lectins. Cytotoxicity towards tumor cells due to the induction of programmed cell death (apoptosis). Arzneimittel-Forschung 1993;43:1221-7.
7. Kovacs E, Link S, Toffol-Schmidt U. Cytostatic and cytocidal effects of mistletoe (Viscum album L.) quercus extract Iscador. Arzneimittel-Forschung 2006;56:467-73.
8. Elluru S, Duong Van Huyen JP, Delignat S, et al. Molecular mechanisms underlying the immunomodulatory effects of mistletoe (Viscum album L.) extracts Iscador. Arzneimittel-Forschung 2006;56:461-6.
9. Hostanska K, Hajto T, Spagnoli GC, Fischer J, Lentzen H, Herrmann R. A plant lectin derived from Viscum album induces cytokine gene expression and protein production in cultures of human peripheral blood mononuclear cells. Natural immunity 1995;14:295-304.
10. Hajto T, Hostanska K, Frei K, Rordorf C, Gabius HJ. Increased secretion of tumor necrosis factors alpha, interleukin 1, and interleukin 6 by human mononuclear cells exposed to beta-galactoside-specific lectin from clinically applied mistletoe extract. Cancer research 1990;50:3322-6.
11. Park WB, Lyu SY, Kim JH, et al. Inhibition of tumor growth and metastasis by Korean mistletoe lectin is associated with apoptosis and antiangiogenesis. Cancer biotherapy & radiopharmaceuticals 2001;16:439-47.
12. Yoon TJ, Yoo YC, Choi OB, et al. Inhibitory effect of Korean mistletoe (Viscum album coloratum) extract on tumour angiogenesis and metastasis of haematogenous and non-haematogenous tumour cells in mice. Cancer letters 1995;97:83-91.
13. Ye W, Nanga RP, Kang CB, Song JH, Song SK, Yoon HS. Molecular characterization of the recombinant A-chain of a type II ribosome-inactivating protein (RIP) from Viscum album coloratum and structural basis on its ribosome-inactivating activity and the sugar-binding properties of the B-chain. Journal of biochemistry and molecular biology 2006;39:560-70.
14. Burkhart J, Walchli C, Heusser P, Weissenstein U, Baumgartner S, Andres AC. In vitro investigation into the potential of a mistletoe extract to alleviate adverse effects of cyclophosphamide. Alternative therapies in health and medicine 2010;16:40-8.
15. Stein GM, Berg PA. Flow cytometric analyses of the specific activation of peripheral blood mononuclear cells from healthy donors after in vitro stimulation with a fermented mistletoe extract and mistletoe lectins. Eur J Cancer 1998;34:1105-10.
16. Mistletoe products from Helixor. 2015. (Accessed at http://www.helixor.com/integrative-cancer-therapy/mistletoe-therapy/products/.)
17. Kienle GS, Kiene H. Complementary cancer therapy: a systematic review of prospective

clinical trials on anthroposophic mistletoe extracts. European journal of medical research 2007;12:103-19.
18. Kienle GS, Kiene H. Review article: Influence of Viscum album L (European mistletoe) extracts on quality of life in cancer patients: a systematic review of controlled clinical studies. Integrative cancer therapies 2010;9:142-57.
19. Melzer J, Iten F, Hostanska K, Saller R. Efficacy and safety of mistletoe preparations (Viscum album) for patients with cancer diseases. A systematic review. Forsch Komplementmed 2009;16:217-26.
20. Friedrichson U. Die Wirkung der Misteltherapie auf Tumorschmerzen [The effects of mistletoe therapy on cancer pain]. Erfahrungsheilkunde 1995;44:669.
21. Kalden M. Klinische Erfahrungen mit Viscum album bei fortgeschrittenen Tumoren [Clinical experience of Viscum album in advanced cancer]. Erfahrungsheilkunde 1994;6:315 – 21.
22. Steinkellner W. Hochdosis-Misteltherapie. Eigendruck 1997:1-3.
23. Sarkadi A. Mistelinfusionstherapie in der Onkologie – eine klinische Verlaufsbeobachtung [Mistletoe infusion therapy in oncology – a clinical report]. Erfahrungsheilkunde 1995;44:426-9.
24. Zerm Rea. Intravenöse Misteltherapie zu Ardalan-Chemotherapie bei einer Patientin mit metastasiertem Kolonkarzinom. [Intravenous mistletoe and the Ardalan chemotherapy regimen in the treatment of a patient suffering from advanced colorectal carcinoma]. Fortschritte in der Misteltherapie KCV Verlag 2005:465-75.
25. Böcher E, Stumpf, C., Büssing, A., Schietzel, M. Prospektive Bewertung der Toxizität hochdosierter Viscum album L.-Infusionen bei Patienten mit progredienten Malignomen [Prospective evaluation of the toxicity of high-dosed Viscum album L. infusions in patients with progressive malignant disease]. Zeitschrift für Onkologie 1996;28:97-106.
26. Salzer G, Hellan, J., Babits, R., Engelhard, I., Günczler, P. Die Mistel am Ludwig-Boltzmann-Institut für klinische Onkologie [The use of mistletoe at the Ludwig Boltzmann Institute for Clinical Oncology]. Deutsche Zeitschrift für Onkologie 1987;19:59-63.
27. Steele ML, Axtner J, Happe A, Kroz M, Matthes H, Schad F. Safety of Intravenous Application of Mistletoe (Viscum album L.) Preparations in Oncology: An Observational Study. Evidence-based complementary and alternative medicine : eCAM 2014;2014:236310.

APPENDIX A: Performance Status Criteria

**ECOG Performance Status Scale**		**Karnofsky Performance Scale**	
Grade	Descriptions	Percent	Description
0	Normal activity. Fully active, able to carry on all pre-disease performance without restriction.	100	Normal, no complaints, no evidence of disease.
		90	Able to carry on normal activity; minor signs or symptoms of disease.
1	Symptoms, but ambulatory. Restricted in physically strenuous activity, but ambulatory and able to carry out work of a light or sedentary nature (e.g., light housework, office work).	80	Normal activity with effort; some signs or symptoms of disease.
		70	Cares for self, unable to carry on normal activity or to do active work.
2	In bed <50% of the time. Ambulatory and capable of all self-care, but unable to carry out any work activities. Up and about more than 50% of waking hours.	60	Requires occasional assistance, but is able to care for most of his/her needs.
		50	Requires considerable assistance and frequent medical care.
3	In bed >50% of the time. Capable of only limited self-care, confined to bed or chair more than 50% of waking hours.	40	Disabled, requires special care and assistance.
		30	Severely disabled, hospitalization indicated. Death not imminent.
4	100% bedridden. Completely disabled. Cannot carry on any self-care. Totally confined to bed or chair.	20	Very sick, hospitalization indicated. Death not imminent.
		10	Moribund, fatal processes progressing rapidly.
5	Dead.	0	Dead.

APPENDIX B: SAE Reporting Form

Serious Adverse Event Reporting Form - Page 1

Please notify within 24 hours: Dr. Paller (Fax: 410-955-5097, email: cpaller1@jhmi.edu)
Christina Raynor (Fax: 410-955-5097, email: craynor1@jhmi.edu)

**Protocol Title:**	A Phase I Dose Escalating Trial of Mistletoe Extract in Patients with Advanced Solid Tumors		
**Protocol Number:**<br><br>J1681	**Principal Investigator:**	**Signature of PI:**	**Date:**
**Report Date:**	**Hospital Admission Date:**<br><br>**Hospital Discharge Date:**	**Date of Discovery of Event:**	**Report Type:**<br>☐**Initial**<br>☐**Follow-up**<br>☐**Final Follow-up**<br>☐**Death**<br>☐**Addendum to:**

Section A: Subject Information

**Subject ID:**	**Subject Initial:**	**Subject Gender:**<br>☐**Male**<br>☐**Female**

Section B: Event Information

**Event diagnosis or symptoms:**	**Date of First Dose:**<br><br>**Date of Last Dose prior to Event:**<br><br>**Number of Total Doses:**	**Action taken with the study drug:**<br>☐**None**<br>☐**Interrupted**<br>☐**Delayed**<br>☐**Discontinued**

**Event Onset Date:**	**Event End Date:**

**Relationship to:**	**Mistletoe**	**Underlying Disease**
**Unrelated**	☐	☐
**Probably Unrelated**	☐	☐
**Possible Related**	☐	☐
**Probably Related**	☐	☐
**Definitely Related**	☐	☐

Serious Adverse Event Reporting Form – Page 2

**Section C: Brief Description of the Event:**

**Section D: Relevant Medical History**

Section E: Concomitant Drug (Not related to SAE)

**Name of the Drug**	**Start Date**	**Stop Date**	**Route**	**Dose**	**Frequency**

**Section F: Comments**
**Additional Documents:** ☐ Please specify

APPENDIX C: FACT-G Questionnaire (Version 4)

Below is a list of statements that other people with your illness have said are important. **Please circle or mark one number per line to indicate your response as it applies to the past 7 days.**

	PHYSICAL WELL-BEING	Not at all	A little bit	Some-what	Quite a bit	Very much
GP1	I have a lack of energy	0	1	2	3	4
GP2	I have nausea	0	1	2	3	4
GP3	Because of my physical condition, I have trouble meeting the needs of my family	0	1	2	3	4
GP4	I have pain	0	1	2	3	4
GP5	I am bothered by side effects of treatment	0	1	2	3	4
GP6	I feel ill	0	1	2	3	4
GP7	I am forced to spend time in bed	0	1	2	3	4

	SOCIAL/FAMILY WELL-BEING	Not at all	A little bit	Some-what	Quite a bit	Very much
GS1	I feel close to my friends	0	1	2	3	4
GS2	I get emotional support from my family	0	1	2	3	4
GS3	I get support from my friends	0	1	2	3	4
GS4	My family has accepted my illness	0	1	2	3	4
GS5	I am satisfied with family communication about my illness	0	1	2	3	4
GS6	I feel close to my partner (or the person who is my main support)	0	1	2	3	4
Q1	*Regardless of your current level of sexual activity, please answer the following question. If you prefer not to answer it, please mark this box ☐ nd go to the next section.*					
GS7	I am satisfied with my sex life	0	1	2	3	4

FACT-G Questionnaire (Version 4) – Page 2

Please circle or mark one number per line to indicate your response as it applies to the past 7 days.

EMOTIONAL WELL-BEING

		Not at all	A little bit	Some-what	Quite a bit	Very much
GE1	I feel sad	0	1	2	3	4
GE2	I am satisfied with how I am coping with my illness	0	1	2	3	4
GE3	I am losing hope in the fight against my illness	0	1	2	3	4
GE4	I feel nervous	0	1	2	3	4
GE5	I worry about dying	0	1	2	3	4
GE6	I worry that my condition will get worse	0	1	2	3	4

FUNCTIONAL WELL-BEING

		Not at all	A little bit	Some-what	Quite a bit	Very much
GF1	I am able to work (include work at home)	0	1	2	3	4
GF2	My work (include work at home) is fulfilling	0	1	2	3	4
GF3	I am able to enjoy life	0	1	2	3	4
GF4	I have accepted my illness	0	1	2	3	4
GF5	I am sleeping well	0	1	2	3	4
GF6	I am enjoying the things I usually do for fun	0	1	2	3	4
GF7	I am content with the quality of my life right now	0	1	2	3	4

APPENDIX D: Response Evaluation Criteria in Solid Tumors (RECIST) 1.1 Criteria for Evaluating Response in Solid Tumors

RECIST version 1.1 will be used in this study for assessment of tumor response. While either CT or MRI may be used utilized, as per RECIST 1.1, CT is the preferred imaging technique in this study.

Disease Parameters

Measurable disease: Measurable lesions are defined as those that can be accurately measured in at least one dimension (longest diameter to be recorded) as ≥20 mm by chest x-ray, as ≥10 mm with CT scan, or ≥10 mm with calipers by clinical exam. All tumor measurements must be recorded in millimeters (or decimal fractions of centimeters).

> Note: Tumor lesions that are situated in a previously irradiated area might or might not be considered measurable unless there is evidence of progression in the irradiated site. Malignant lymph nodes. To be considered pathologically enlarged and measurable, a lymph node must be ≥15 mm in short axis when assessed by CT scan (CT scan slice thickness recommended to be no greater than 5 mm). At baseline and in follow-up, only the short axis will be measured and followed.

Non-measurable disease: All other lesions (or sites of disease), including small lesions (longest diameter <10 mm or pathological lymph nodes with ≥10 to <15 mm short axis), are considered non-measurable disease. Bone lesions, leptomeningeal disease, ascites, pleural/pericardial effusions, lymphangitis cutis/pulmonitis, inflammatory breast disease, and abdominal masses (not followed by CT or MRI), are considered as non-measurable.

> Note: Cystic lesions that meet the criteria for radiographically defined simple cysts should not be considered as malignant lesions (neither measurable nor non-measurable) since they are, by definition, simple cysts.
>
> 'Cystic lesions' thought to represent cystic metastases can be considered as measurable lesions, if they meet the definition of measurability described above. However, if non-cystic lesions are present in the same patient, these are preferred for selection as target lesions.

Target lesions: All measurable lesions up to a maximum of 2 lesions per organ and 5 lesions in total, representative of all involved organs, should be identified as **target lesions** and recorded and measured at baseline. Target lesions should be selected on the basis of their size (lesions with the longest diameter), be representative of all involved organs, but in addition should be those that lend themselves to reproducible repeated measurements. It may be the case that, on occasion, the largest lesion does not lend itself to reproducible measurement in which circumstance the next largest lesion which can be measured reproducibly should be selected. A sum of the diameters (longest for non-nodal lesions, short axis for nodal lesions) for all target lesions will be calculated and reported as the baseline sum diameters. If lymph nodes are to be included in the sum, then only the short axis is added into the sum. The baseline sum diameters will be used as reference to further characterize any objective tumor regression in the measurable dimension of the disease.

Non-target lesions: All other lesions (or sites of disease) including any measurable lesions over

and above the 5 target lesions should be identified as **non-target lesions** and should also be recorded at baseline. Measurements of these lesions are not required, but the presence, absence, or in rare cases unequivocal progression of each should be noted throughout follow-up.

Evaluation of Target Lesions

Complete Response (CR): Disappearance of all target lesions. Any pathological lymph nodes (whether target or non-target) must have reduction in short axis to <10 mm.

Partial Response (PR): At least a 30% decrease in the sum of the diameters of target lesions, taking as reference the baseline sum diameters.

Progressive Disease (PD): At least a 20% increase in the sum of the diameters of target lesions, taking as reference the smallest sum on study (this includes the baseline sum if that is the smallest on study). In addition to the relative increase of 20%, the sum must also demonstrate an absolute increase of at least 5 mm. (Note: the appearance of one or more new lesions is also considered progressions).

Stable Disease (SD): Neither sufficient shrinkage to qualify for PR nor sufficient increase to qualify for PD, taking as reference the smallest sum diameters while on study.

Evaluation of Non-Target Lesions

Complete Response (CR): Disappearance of all non-target lesions and normalization of tumor marker level. All lymph nodes must be non-pathological in size (<10 mm short axis).

Note: If tumor markers are initially above the upper normal limit, they must normalize for a patient to be considered in complete clinical response.

Non-CR/Non-PD: Persistence of one or more non-target lesion(s) and/or maintenance of tumor marker level above the normal limits.

Progressive Disease (PD): Appearance of one or more new lesions and/or *unequivocal progression* of existing non-target lesions. *Unequivocal progression* should not normally trump target lesion status. It must be representative of overall disease status change, not a single lesion increase.

Although a clear progression of "non-target" lesions only is exceptional, the opinion of the treating physician should prevail in such circumstances, and the progression status should be confirmed at a later time by the review panel (or Principal Investigator).

Evaluation of Best Overall Response

The best overall response is the best response recorded from the start of the treatment until disease progression/recurrence (taking as reference for progressive disease the smallest measurements recorded since the treatment started). The patient's best response assignment will depend on the achievement of both measurement and confirmation criteria.

For Patients with Measurable Disease (i.e., Target Disease)

Target Lesions	Non-Target Lesions	New Lesions	Overall Response	Best Overall Response when Confirmation is Required*
CR	CR	No	CR	≥4 wks. Confirmation**
CR	Non-CR/Non-PD	No	PR	≥4 wks. Confirmation**
CR	Not evaluated	No	PR	
PR	Non-CR/Non-PD/not evaluated	No	PR	
SD	Non-CR/Non-PD/not evaluated	No	SD	Documented at least once ≥4 wks. from baseline**
PD	Any	Yes or No	PD	no prior SD, PR or CR
Any	PD***	Yes or No	PD	
Any	Any	Yes	PD	

* See RECIST 1.1 manuscript for further details on what is evidence of a new lesion.

** Only for non-randomized trials with response as primary endpoint.

*** In exceptional circumstances, unequivocal progression in non-target lesions may be accepted as disease progression.

Note: Patients with a global deterioration of health status requiring discontinuation of treatment without objective evidence of disease progression at that time should be reported as "*symptomatic deterioration.*" Every effort should be made to document the objective progression even after discontinuation of treatment.

Reference

E.A. Eisenhauer, P. Therasse, J. Bogaerts, L.H. Schwartz, D. Sargent, R. Ford, J. Dancey, S. Arbuck, S. Gwyther, M. Mooney, L. Rubinstein, L. Shankar, L. Dodd, R. Kaplan, D. Lacombe, J. Verweij. New response evaluation criteria in solid tumours: Revised RECIST guideline (version 1.1). Eur J Cancer. 2009 Jan;45(2):228-47.

APPENDIX E: Immune Related Response Criteria (irRC)

For all patients who experience disease progression on study, the date noted for of disease progression is the time of the scan where it is originally detected, and not the following date of the confirmatory scan.

Definitions of measurable and non-measurable disease

Measurable disease: Neoplastic masses that can be precisely measured in 2 in-plane perpendicular diameters. Both its longest diameter and its longest perpendicular must be greater than or equal to 10 mm. Lymph nodes must have a short-axis line-length of ≥ 15 mm. Malignant lymph nodes must be measurable in 2 perpendicular diameters. Both its longest diameter and its longest perpendicular must be greater than or equal to 15 mm. The quantitative endpoint will be defined as the product of the longest diameter with its longest perpendicular.

Non-measurable disease: Non-measurable lesions are those that are not suitable for quantitative assessment over time. These include:

1) Neoplastic masses that are too small to measure, because their longest uninterrupted diameter or longest perpendicular are less than 10 mm.

2) Neoplastic masses whose boundaries cannot be distinguished. This includes masses which cannot be demarcated from surrounding tissue because of inadequate contrast, masses with overly complex morphology, or those with highly heterogeneous tissue composition.

3) Other types of lesions that are confidently felt to represent neoplastic tissue, but difficult to quantify in a reproducible manner. These include bone metastases, leptomeningeal metastases, malignant ascites, pleural/pericardial effusions, inflammatory breast disease, lymphangitis cutis/pulmonis, cystic lesions, ill-defined abdominal masses, etc.

For irRC, only target lesions selected at baseline and measurable new lesions are taken into account.

At the baseline tumor assessment, the sum of the products of the two largest perpendicular diameters (SPD) of all **index lesions** (five lesions per organ, up to 10 visceral lesions and five cutaneous index lesions) is calculated.

At each subsequent tumor assessment, the SPD of the index lesions and of new, measurable lesions (≥ 5 X 5 mm; up to 5 new lesions per organ: 5 new cutaneous lesions and 10 visceral lesions) are added together to provide the total time-point **tumor burden.**

Overall response using irRC:

- **Complete Response (irCR):** Complete disappearance of all tumor lesions (whether measureable or not, and no new lesions). CR must be confirmed by repeated, consecutive assessments made no less than 4 weeks from the date first documented.
- **Partial Response (irPR):** Decrease in SPD of 50% or greater by a consecutive assessment at least 4 weeks after first documentation.
- **Stable Disease (irSD):** Failure to meet criteria for irCR or irPR, in absence of irPD.
- **Progressive Disease (irPD):** At least 25% increase in SPD relative to nadir (minimum recorded tumor burden) Confirmation by a repeat, consecutive assessment no less than 4 weeks from the data first documented.

Please note other key differences between irRC and the original WHO criteria:

New measurable lesions will be incorporated into the SPD

New non measurable lesions do not define progression but preclude irCR

Non-index lesions contribute to defining irCR (complete disappearance required).

See the Investigators Imaging Operations Manual (IIOM) for more details.

REFERENCE

IrRC for the current protocol is adopted from the following reference:

Wolchok, JD, Hoos, A, O'Day S, et al., Guidelines for the Evaluation of Immune Therapy Activity in Solid Tumors: Immune-Related Response Criteria. Clinical Cancer Research, 2009 Dec 1;15(23):7412-20. Epub 2009 Nov 24.
