# Supplementary material for: Phase I Trial of Intravenous Mistletoe Extract in Advanced Cancer
Source: Cancer Res Commun. 2023 Feb 28;3(2):338–46. doi: 10.1158/2767-9764.CRC-23-0002 (PMC9973409; doi:10.1158/2767-9764.CRC-23-0002)
Supplement: Table ST1 — Table S1 shows summary of all adverse events [file crc-23-0002-s02.docx]

# Table S1. Summary of All Adverse Events (N=21, any grade incidence 5% or lower)

|  | Any Grade | Grade 1 | Grade 2 | Grade 3 | Grade 4 |
| --- | --- | --- | --- | --- | --- |
| Any events | 20(95.2%) | 2(15%) | 7(35%) | 8(40%) | 2(10%) |
| Abdominal distension | 1(4.8%) | 1(4.8%) | 0(0%) | 0(0%) | 0(0%) |
| Alanine aminotransferase increased | 1(4.8%) | 0(0%) | 0(0%) | 1(4.8%) | 0(0%) |
| Anxiety | 1(4.8%) | 0(0%) | 0(0%) | 1(4.8%) | 0(0%) |
| Arthralgia | 1(4.8%) | 1(4.8%) | 0(0%) | 0(0%) | 0(0%) |
| Arthritis | 1(4.8%) | 1(4.8%) | 0(0%) | 0(0%) | 0(0%) |
| Aspartate aminotransferase increased | 1(4.8%) | 0(0%) | 1(4.8%) | 0(0%) | 0(0%) |
| Atrial fibrillation | 1(4.8%) | 0(0%) | 0(0%) | 0(0%) | 1(4.8%) |
| Bloating | 1(4.8%) | 1(4.8%) | 0(0%) | 0(0%) | 0(0%) |
| Blood bilirubin increased | 1(4.8%) | 0(0%) | 1(4.8%) | 0(0%) | 0(0%) |
| Chest wall pain | 1(4.8%) | 1(4.8%) | 0(0%) | 0(0%) | 0(0%) |
| Cough | 1(4.8%) | 1(4.8%) | 0(0%) | 0(0%) | 0(0%) |
| Duodenal hemorrhage | 1(4.8%) | 0(0%) | 0(0%) | 1(4.8%) | 0(0%) |
| Dysphagia | 1(4.8%) | 1(4.8%) | 0(0%) | 0(0%) | 0(0%) |
| Edema trunk | 1(4.8%) | 1(4.8%) | 0(0%) | 0(0%) | 0(0%) |
| Fever | 1(4.8%) | 1(4.8%) | 0(0%) | 0(0%) | 0(0%) |
| Flatulence | 1(4.8%) | 1(4.8%) | 0(0%) | 0(0%) | 0(0%) |
| Flu like symptoms | 1(4.8%) | 1(4.8%) | 0(0%) | 0(0%) | 0(0%) |
| General disorders-admin site condition | 1(4.8%) | 1(4.8%) | 0(0%) | 0(0%) | 0(0%) |
| Genital edema | 1(4.8%) | 0(0%) | 1(4.8%) | 0(0%) | 0(0%) |
| Hot flashes | 1(4.8%) | 1(4.8%) | 0(0%) | 0(0%) | 0(0%) |
| Hyperhidrosis | 1(4.8%) | 1(4.8%) | 0(0%) | 0(0%) | 0(0%) |
| Hypokalemia | 1(4.8%) | 1(4.8%) | 0(0%) | 0(0%) | 0(0%) |
| Hyponatremia | 1(4.8%) | 0(0%) | 0(0%) | 1(4.8%) | 0(0%) |
| Hypophosphatemia | 1(4.8%) | 1(4.8%) | 0(0%) | 0(0%) | 0(0%) |
| Hypoxia | 1(4.8%) | 0(0%) | 0(0%) | 1(4.8%) | 0(0%) |
| Immune system disorders | 1(4.8%) | 0(0%) | 0(0%) | 1(4.8%) | 0(0%) |
| Infections and infestations | 1(4.8%) | 0(0%) | 0(0%) | 1(4.8%) | 0(0%) |
| Infusion related reaction | 1(4.8%) | 0(0%) | 1(4.8%) | 0(0%) | 0(0%) |
| INR increased | 1(4.8%) | 1(4.8%) | 0(0%) | 0(0%) | 0(0%) |
| Infestations | 1(4.8%) | 1(4.8%) | 0(0%) | 0(0%) | 0(0%) |
| Lip infection | 1(4.8%) | 0(0%) | 1(4.8%) | 0(0%) | 0(0%) |
| Mucositis oral | 1(4.8%) | 0(0%) | 1(4.8%) | 0(0%) | 0(0%) |
| Myalgia | 1(4.8%) | 1(4.8%) | 0(0%) | 0(0%) | 0(0%) |
| Paresthesia | 1(4.8%) | 1(4.8%) | 0(0%) | 0(0%) | 0(0%) |
| Pericardial effusion | 1(4.8%) | 0(0%) | 0(0%) | 1(4.8%) | 0(0%) |
| Peripheral motor neuropathy | 1(4.8%) | 0(0%) | 1(4.8%) | 0(0%) | 0(0%) |
| Pleuritic pain | 1(4.8%) | 0(0%) | 1(4.8%) | 0(0%) | 0(0%) |
| Productive cough | 1(4.8%) | 0(0%) | 1(4.8%) | 0(0%) | 0(0%) |
| Pruritus | 1(4.8%) | 1(4.8%) | 0(0%) | 0(0%) | 0(0%) |
| Rash maculo-papular | 1(4.8%) | 1(4.8%) | 0(0%) | 0(0%) | 0(0%) |
| Scrotal pain | 1(4.8%) | 0(0%) | 1(4.8%) | 0(0%) | 0(0%) |
| Sneezing | 1(4.8%) | 1(4.8%) | 0(0%) | 0(0%) | 0(0%) |
| Sore throat | 1(4.8%) | 1(4.8%) | 0(0%) | 0(0%) | 0(0%) |
| Thromboembolic event | 1(4.8%) | 0(0%) | 0(0%) | 1(4.8%) | 0(0%) |
| Urinary tract infection | 1(4.8%) | 0(0%) | 1(4.8%) | 0(0%) | 0(0%) |
| Urinary tract pain | 1(4.8%) | 0(0%) | 1(4.8%) | 0(0%) | 0(0%) |
| Vaginal hemorrhage | 1(4.8%) | 1(4.8%) | 0(0%) | 0(0%) | 0(0%) |
| Vascular disorders | 1(4.8%) | 1(4.8%) | 0(0%) | 0(0%) | 0(0%) |
