# Supplementary material for: Phase I Trial of Intravenous Mistletoe Extract in Advanced Cancer
Source: Cancer Res Commun. 2023 Feb 28;3(2):338–46. doi: 10.1158/2767-9764.CRC-23-0002 (PMC9973409; doi:10.1158/2767-9764.CRC-23-0002)
Supplement: Table ST2 — Table S2 shows treatment-related adverse events by dose level [file crc-23-0002-s03.docx]

# Table S2. Treatment-related Adverse Events by Dose Level

|  | **150 mg (N=8)** | | **300 mg (N=3)** | | **600 mg (N=8)** | | **900 mg (N=2)** | | **All (N=21)** | |
| --- | --- | --- | --- | --- | --- | --- | --- | --- | --- | --- |
|  | **All grades** | **Grade 3** | **All grades** | **Grade 3** | **All grades** | **Grade 3** | **All grades** | **Grade 3** | **All grades** | **Grade 3** |
| Any events | 5 (62.5%) | 1 (12.5%) | 2 (66.7%) | 0 (0%) | 4 (50%) | 0 (0%) | 2 (100%) | 2 (100%) | 13 (61.9%) | 3 (14.3%) |
| Abdominal pain | 1 (12%) | 0 (0%) | 0 (0%) | 0 (0%) | 0 (0%) | 0 (0%) | 0 (0%) | 0 (0%) | 1 (4.8%) | 0 (0%) |
| Alanine aminotransferase | 0 (0%) | 0 (0%) | 0 (0%) | 0 (0%) | 0 (0%) | 0 (0%) | 1 (50%) | 1 (50%) | 1 (4.8%) | 1 (4.8%) |
| Arthritis | 1 (12%) | 0 (0%) | 0 (0%) | 0 (0%) | 0 (0%) | 0 (0%) | 0 (0%) | 0 (0%) | 1 (4.8%) | 0 (0%) |
| Chills | 1 (12%) | 0 (0%) | 0 (0%) | 0 (0%) | 1 (12.5%) | 0 (0%) | 0 (0%) | 0 (0%) | 2 (9.5%) | 0 (0%) |
| Cough | 0 (0%) | 0 (0%) | 0 (0%) | 0 (0%) | 1 (12.5%) | 0 (0%) | 0 (0%) | 0 (0%) | 1 (4.8%) | 0 (0%) |
| Diarrhea | 1 (12%) | 0 (0%) | 0 (0%) | 0 (0%) | 0 (0%) | 0 (0%) | 0 (0%) | 0 (0%) | 1 (4.8%) | 0 (0%) |
| Dyspnea | 0 (0%) | 0 (0%) | 0 (0%) | 0 (0%) | 0 (0%) | 0 (0%) | 1 (50%) | 1 (50%) | 1 (4.8%) | 1 (4.8%) |
| Edema | 2(24%) | 0 (0%) | 0 (0%) | 0 (0%) | 0 (0%) | 0 (0%) | 0 (0%) | 0 (0%) | 2(9.6%) | 0 (0%) |
| Fatigue | 3 (37.5%) | 1 (12.5%) | 1 (33%) | 0 (0%) | 2 (25%) | 0 (0%) | 0 (0%) | 0 (0%) | 6 (28.%) | 1 (4.8%) |
| Flank pain | 0 (0%) | 0 (0%) | 0 (0%) | 0 (0%) | 0 (0%) | 0 (0%) | 1 (50%) | 1 (50%) | 1 (4.8%) | 1 (4.8%) |
| Flu-like symptoms | 0 (0%) | 0 (0%) | 1 (33%) | 0 (0%) | 0 (0%) | 0 (0%) | 0 (0%) | 0 (0%) | 1 (4.8%) | 0 (0%) |
| Hot flashes | 1 (12%) | 0 (0%) | 0 (0%) | 0 (0%) | 0 (0%) | 0 (0%) | 0 (0%) | 0 (0%) | 1 (4.8%) | 0 (0%) |
| Myalgia | 1 (12%) | 0 (0%) | 0 (0%) | 0 (0%) | 0 (0%) | 0 (0%) | 0 (0%) | 0 (0%) | 1 (4.8%) | 0 (0%) |
| Nausea | 1 (12%) | 0 (0%) | 0 (0%) | 0 (0%) | 1 (12.5%) | 0 (0%) | 0 (0%) | 0 (0%) | 2 (9.5%) | 0 (0%) |
| Pain | 1 (12%) | 0 (0%) | 0 (0%) | 0 (0%) | 0 (0%) | 0 (0%) | 0 (0%) | 0 (0%) | 1 (4.8%) | 0 (0%) |
| Peeling skin (soles of feet) | 0 (0%) | 0 (0%) | 0 (0%) | 0 (0%) | 0 (0%) | 0 (0%) | 1 (50%) | 0 (0%) | 1 (4.8%) | 0 (0%) |
| Pleuritic pain | 0 (0%) | 0 (0%) | 0 (0%) | 0 (0%) | 0 (0%) | 0 (0%) | 1 (50%) | 0 (0%) | 1 (4.8%) | 0 (0%) |
| Pruritus | 1 (12%) | 0 (0%) | 0 (0%) | 0 (0%) | 0 (0%) | 0 (0%) | 0 (0%) | 0 (0%) | 1 (4.8%) | 0 (0%) |
| Rash acneiform | 1 (12%) | 0 (0%) | 0 (0%) | 0 (0%) | 0 (0%) | 0 (0%) | 0 (0%) | 0 (0%) | 1 (4.8%) | 0 (0%) |
| Sneezing | 0 (0%) | 0 (0%) | 0 (0%) | 0 (0%) | 1 (12.5%) | 0 (0%) | 0 (0%) | 0 (0%) | 1 (4.8%) | 0 (0%) |
