# Supplementary material for: Phase I Trial of Intravenous Mistletoe Extract in Advanced Cancer
Source: Cancer Res Commun. 2023 Feb 28;3(2):338–46. doi: 10.1158/2767-9764.CRC-23-0002 (PMC9973409; doi:10.1158/2767-9764.CRC-23-0002)
Supplement: Table ST3 — Table S3 shows reasons for discontinuation with patient charateristics [file crc-23-0002-s04.docx]

# Table S3. Reasons for Discontinuation with Patient Characteristics

| **Patient** | **Gender** | **Race** | **Tumor type (histology)** | **Prior lines of therapy** | **Date of last dose** | **Dose level** | **Reason for**  **discontinuation** |
| --- | --- | --- | --- | --- | --- | --- | --- |
| JHU-001 | F | White | Neuroendocrine (carcinoid) | 3 | W1-D3 | 150mg | Treatment toxicity |
| JHU-002 | F | White | Colon (adenocarcinoma) | 4 | W7-D2 | 150mg | Clinical progression |
| JHU-003 | F | White | Ovarian (invasive low grade serous carcinoma) | 11 | W8-D1 | 150mg | Progressive disease |
| JHU-004 | F | Black/African American | Pancreatic (adenocarcinoma) | 3 | W3-D2 | 150mg | Patient withdrawal |
| JHU-005 | F | White | Colon (adenocarcinoma) | 2 | W7-D3 | 150mg | Clinical progression |
| JHU-006 | F | White | Synovial sarcoma (biphasic) | 1 | W7-D3 | 150mg | Progressive disease |
| JHU-007 | M | White | Colon (carcinoma) | 2 | W7-D3 | 150mg | Progressive disease |
| JHU-008 | F | White | Salivary gland (polymorphous adenocarcinoma) | 1 | W13-D1 | 150mg | Patient withdrawal |
| JHU-009 | M | Other | Rectal (adenocarcinoma) | 3 | W6-D1 | 300mg | Clinical progression |
| JHU-010 | F | White | Uterine leiomyosarcoma (mixed spindle cell and epithelioid) | 2 | W7-D3 | 300mg | Progressive disease |
| JHU-011 | F | White | Ovarian (high grade serous carcinoma) | 6 | W13-D1 | 300mg | Clinical progression |
| JHU-012 | F | White | Goblet cell (adenocarcinoma ex-goblet carcinoid) | 3 | W24-D2 | 600mg | Patient withdrawal |
| JHU-013 | M | Unknown | Pancreas (adenocarcinoma moderately to poorly differentiated) | 1 | W3-D3 | 600mg | Progressive disease |
| JHU-014 | F | Unknown | Colon (adenocarcinoma) | 2 | W2-D2 | 600mg | Progressive disease |
| JHU-015 | M | Other | Rectal (adenocarcinoma) | 2 | W7-D3 | 600mg | Progressive disease |
| JHU-016 | F | Black/African American | Colon (invasive moderately differentiated adenocarcinoma) | 2 | W3-D3 | 900mg | Treatment toxicity |
| JHU-017 | M | Black/African American | Breast (infiltrating ductal carcinoma moderately differentiated) | 7 | W2-D1 | 900mg | Treatment toxicity |
| JHU-018 | F | White | Basal cell (carcinoma nodular with ulcerage) | 1 | W6-D1 | 600mg | Progressive disease |
| JHU-019 | M | White | Melanoma (unknown) | 1 | W1-D3 | 600mg | Clinical progression |
| JHU-020 | M | White | Lung (squamous cell) | 4 | W4-D3 | 600mg | Progressive disease |
| JHU-021 | F | Black/African American | Ovarian (high grade serous adenocarcinoma) | 4 | W5-D3 | 600mg | Clinical progression |
