# Supplementary material for: Phase I Trial of Intravenous Mistletoe Extract in Advanced Cancer
Source: Cancer Res Commun. 2023 Feb 28;3(2):338–46. doi: 10.1158/2767-9764.CRC-23-0002 (PMC9973409; doi:10.1158/2767-9764.CRC-23-0002)
Supplement: Figure S2 — shows summary of normalized serum levels of cytokins/chemokines/growth factors at week 4 [file crc-23-0002-s06.docx]

# Figure S2. Summary of Baseline Normalized Serum Levels of Cytokines/Chemokines/Growth Factors at Week 4

**
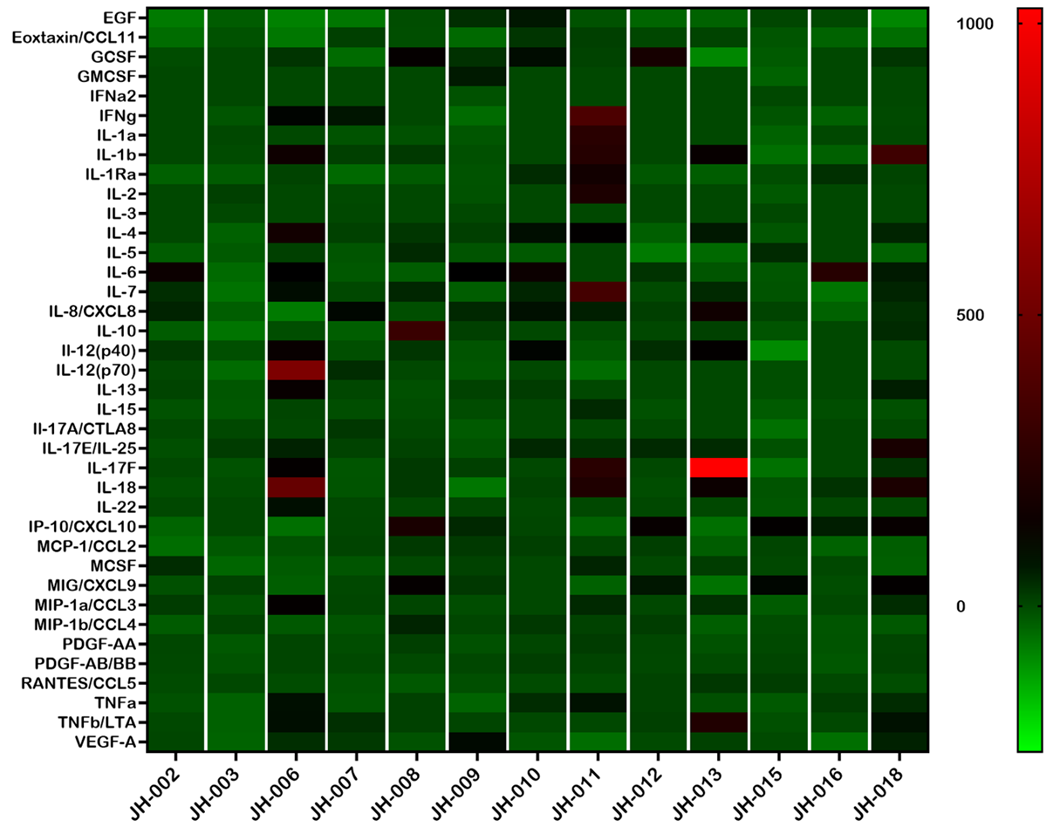
**

Note: This heat map presents a summary of baseline normalized cytokine, chemokine, and growth factor levels in various subjects at a 4-week time point. Levels were normalized to baseline values, and differential values were used to generate the plot. Serum samples were analyzed using Milliplex assay (Millipore), and the concentration of specific analyte recorded was normalized to the respective baseline value to obtain the differential level. Six patients (JHU-001, 004, 005, 014, 017, 020) were also excluded because only baseline samples for them were available to include in the analysis and patient JHU-021 was also excluded because only the week 8 sample was available.
