# Supplementary material for: Phase I Trial of Intravenous Mistletoe Extract in Advanced Cancer
Source: Cancer Res Commun. 2023 Feb 28;3(2):338–46. doi: 10.1158/2767-9764.CRC-23-0002 (PMC9973409; doi:10.1158/2767-9764.CRC-23-0002)
Supplement: Figure S3 — shows functional assessment of cancer therapy - general over time [file crc-23-0002-s07.docx]

# Figure S3. Functional Assessment of Cance Therapy – General Over Time


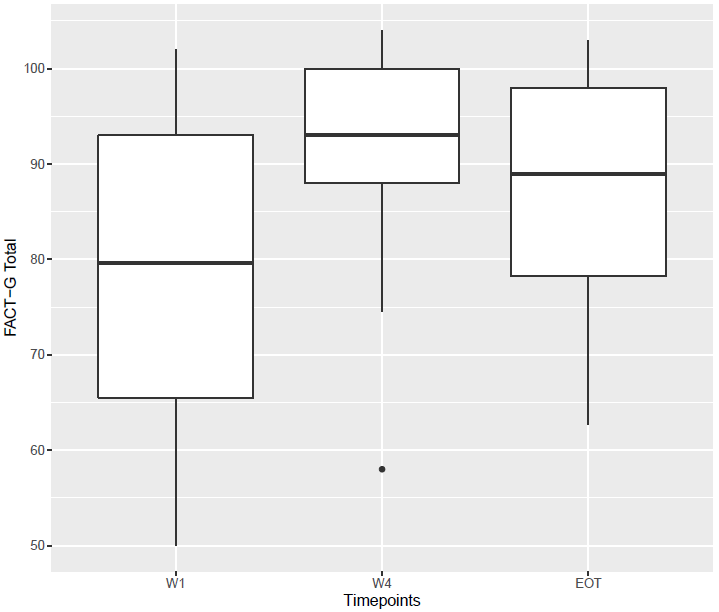


Abbreviations: EOT: end of treatment; FACT-G: Functional Assessment of Cancer Therapy – General; W1: week1; W4: week4.
