# Supplementary material for: Phase I Trial of Intravenous Mistletoe Extract in Advanced Cancer
Source: Cancer Res Commun. 2023 Feb 28;3(2):338–46. doi: 10.1158/2767-9764.CRC-23-0002 (PMC9973409; doi:10.1158/2767-9764.CRC-23-0002)
Supplement: Figure S7 — shows social/family well-being over time [file crc-23-0002-s11.docx]

# Figure S7. Social/Family Well-Being Over Time


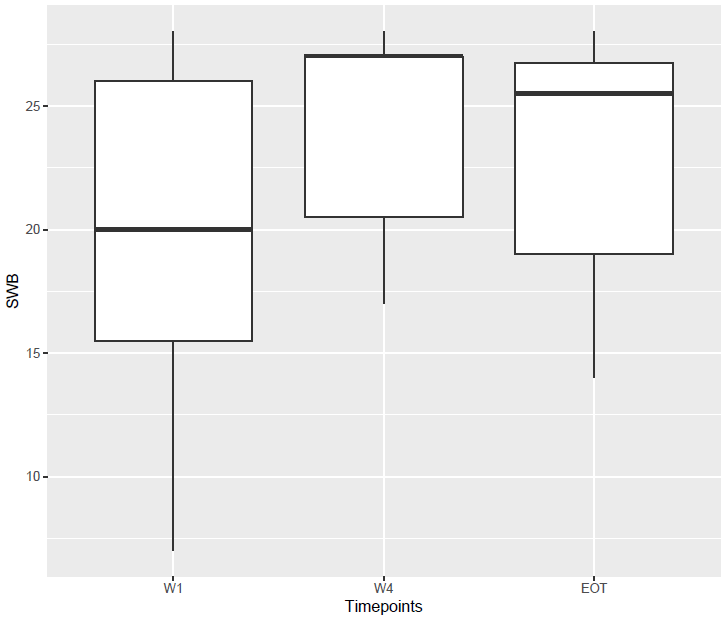


Abbreviations: EOT: end of treatment; SWB: social/family well-being; W1: week 1; W4: week 4. Abbreviations: EOT: end of treatment; PD: progressive disease; PWB: physical well-being; SD: stable disease; W1: week 1; W4: week 4.
