# Supplementary material for: Phase I Trial of Intravenous Mistletoe Extract in Advanced Cancer
Source: Cancer Res Commun. 2023 Feb 28;3(2):338–46. doi: 10.1158/2767-9764.CRC-23-0002 (PMC9973409; doi:10.1158/2767-9764.CRC-23-0002)
Supplement: Figure S9 — shows emotional well-being over time [file crc-23-0002-s13.docx]

# Figure S9. Emotional Well-Being Over Time


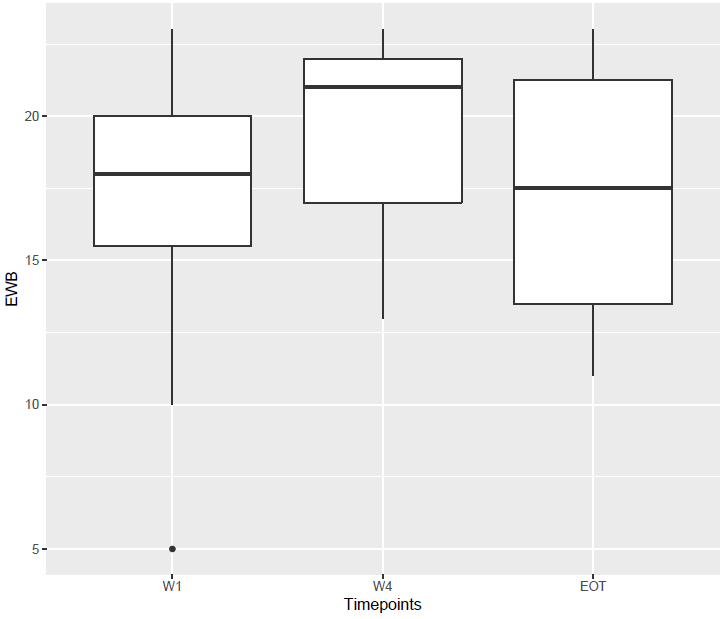


Abbreviations: EOT: end of treatment; EWB: emotional well-being; W1: week 1; W4: week 4.
