# Supplementary material for: Phase I Trial of Intravenous Mistletoe Extract in Advanced Cancer
Source: Cancer Res Commun. 2023 Feb 28;3(2):338–46. doi: 10.1158/2767-9764.CRC-23-0002 (PMC9973409; doi:10.1158/2767-9764.CRC-23-0002)
Supplement: Figure S12 — shows functional well-being over time by response [file crc-23-0002-s16.docx]

# Figure S12. Functional Well-Being Over Time by Response


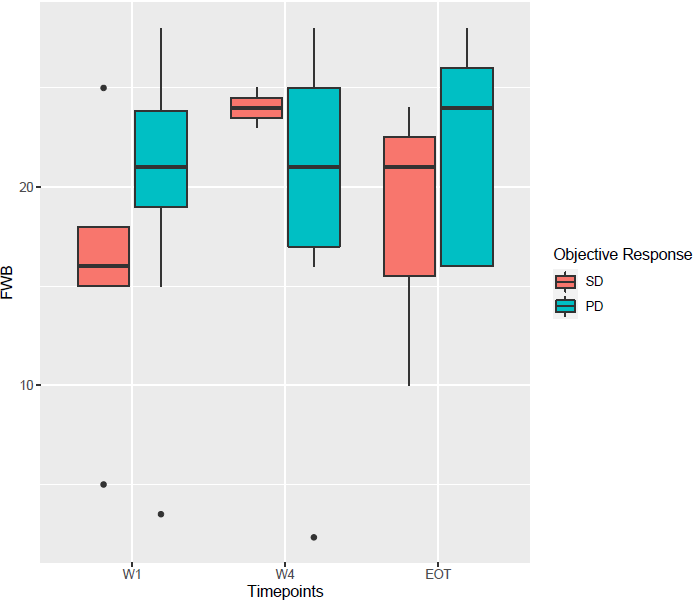


Abbreviations: EOT: end of treatment; FWB: functional well-being; PD: progressive disease; SD: stable disease; W1: week 1; W4: week 4.
